# The crystal engineering of radiation-sensitive diacetylene cocrystals and salts†

**DOI:** 10.1039/d0sc02540b

**Published:** 2020-07-20

**Authors:** Amy V. Hall, Dmitry S. Yufit, David C. Apperley, Larry Senak, Osama M. Musa, David K. Hood, Jonathan W. Steed

**Affiliations:** Durham University, Department of Chemistry Lower Mountjoy, Stockton Road Durham DH1 3LE UK jon.steed@durham.ac.uk; Ashland LLC 1005 Route 202/206 Bridgewater NJ 08807 USA

## Abstract

In this work we develop photoreactive cocrystals/salts of a commercially-important diacetylene, 10,12-pentacosadiynoic acid (PCDA, **1**) and report the first X-ray crystal structures of PCDA based systems. The topochemical reactivity of the system is modified depending on the coformer used and correlates with the structural parameters. Crystallisation of **1** with 4,4′-azopyridine (**2**), 4,4′-bipyridyl (**3**), and *trans*-1,2-bis(4-pyridyl)ethylene (**4**) results in unreactive 2 : 1 cocrystals or a salt in the case of 4,4′-bipiperidine (**5**). However, salt formation with morpholine (**6**), diethylamine (**7**), and *n*-butylamine (**8**), results in highly photoreactive salts **12·7** and **1·8** whose reactivity can be explained using topochemical criteria. The salt **1·6** is also highly photoreactive and is compared to a model morpholinium butanoate salt. Resonance Raman spectroscopy reveals structural details of the photopolymer including its conformational disorder in comparison to less photoactive alkali metal salts and the extent of solid state conversion can be monitored by CP-MAS NMR spectroscopy. We also report an unusual catalysis in which amine evaporation from photopolymerised PCDA ammonium salts effectively acts as a catalyst for polymerisation of PCDA itself. The new photoreactive salts exhibit more reactivity but decreased conjugation compared to the commercial lithium salt and are of considerable practical potential in terms of tunable colours and greater range in UV, X-ray, and γ-ray dosimetry applications.

## Abstract 2

In this work we develop photoreactive cocrystals/salts of a commercially-important diacetylene, 10,12-pentacosadiynoic acid (PCDA, **1**) and report the first X-ray crystal structures of PCDA based systems.

## Graphical abstract

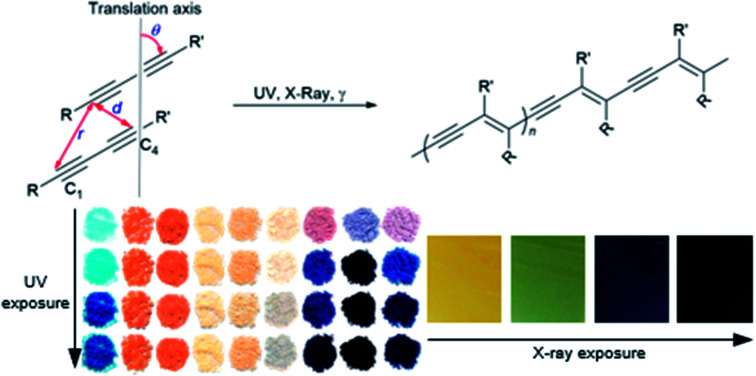

## Introduction

**Scheme 1:**
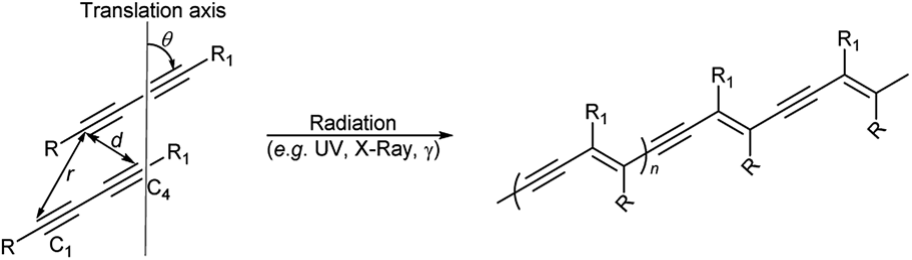
The topochemical parameters for diacetylene 1,4-addition polymerisation requires the tilt angle (*θ*) of the monomers to be 45°, the C1–C4′ distance (*r*) to be ≤3.8 Å, and the translational repeat distance (*d*) to be ≤4.9 Å to yield a polydiacetylene.

The solid state 1,4-addition polymerisation of diacetylenes can be initiated by radiation and heat, and results in a conjugated ene–yne polymeric chain. The reaction is thought to only occur if the topochemical parameters of the diacetylene packing are optimal. The first report of topochemical reactivity in the solid state was in an alkene system described by Schmidt in 1964 who suggested that carbon double bonds must be separated by a maximum distance of 4.2 Å for successful polymerisation.^1^ In 1969, Wegner reported the first example of diacetylene polymerisation in the solid state,^2,3^ while 15 years later, Enkelmann proposed strict criteria for diacetylene reactivity, whereby adjacent diacetylene monomers will react when the reactive groups are separated by a C1–C4′ contact distance (*d*) of ≤3.8 Å, a translational period repeat spacing (*r*) of ≤4.9 Å, along with an orientation angle (*θ*) to the crystal axis at an optimum value of 45° (Scheme 1).^4–6^ The diacetylene polymerisation parameters highlight the importance of molecular organisation in the topochemical reaction.^7,8^

**Scheme 2:**
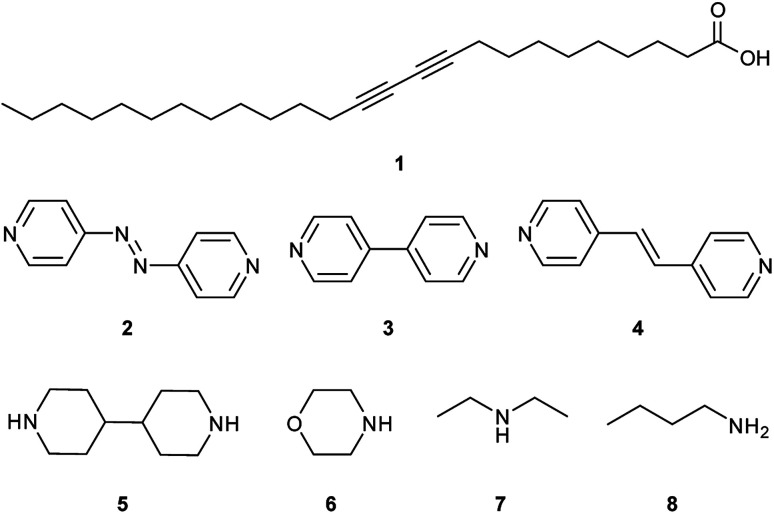
Structures of PCDA (**1**), with coformers 4,4′-azopyridine (**2**), 4,4′-bipyridyl (**3**), *trans*-1,2-bis(4-pyridyl)ethylene (**4**), and salt formers 4,4′-bipiperidine (**5**), morpholine (**6**), diethylamine (**7**) and *n*-butylamine (**8**).

The monomer-to-polymer transition is clearly observed by a colour change from colourless to blue, due to the rearrangement of the diacetylene monomers to give an ene–yne chromophore. The blue colour is due to π–π* transitions in the ordered, conjugated chain with the reorganisation of the chains controlling the degree of diacetylene polymerisation.^9^ Additional external stimuli on the polymerised diacetylene (polydiacetylene) such as extended heating,^10–12^ pH change,^10,13–16^ treatment with organic solvents,^10,17–21^ mechanical stress,^22,23^ and ligand–receptor interactions,^24,25^ can cause the polydiacetylenes to exhibit a range of colours from blue, to red, to yellow.^15^ These chromic changes can be explained by a conformational rearrangement within the polydiacetylene assembly which disrupts the conjugated backbone, causing reduced overlap of the π orbitals, resulting in a widening of the HOMO–LUMO energy gap and hence the polydiacetylene absorbing light at a higher energy.^12,26,27^ The commercially important diacetylene, 10,12-pentacosadiynoic acid (PCDA, **1**), is used to provide a colourimetric change in practical chemosensors,^28–33^ biosensors,^24,34–36^ and dosimeters.^37–40^ Although PCDA is somewhat photoreactive, further tuning of its photoresponse is of considerable interest, especially for radiation dosimetry applications. Covalent modification offers a viable strategy to PCDA analogues with a tuned photoresponse.^41–43^ However, since the solid state reactivity of dialkynes depends on their crystal packing arrangement, a simpler strategy is to address the dialkyne reactivity through modification of non-covalent interactions by cocrystal or salt formation.^44–47^ Whether a cocrystal or salt will form depends on the difference in p*K*_a_ of the two components. For a cocrystal, the Δp*K*_a_ must be <2–3 log units, while salt formation is expected for a greater difference.^48,49^ Cocrystals of carboxylic acids can be prepared using the robust hydrogen-bonded COOH⋯N_pyridine_ heterosynthon, while salts can be based on ammonium complexes of more basic amines.^50–54^ In this work we explore the relationship between structure and photochemistry for PCDA (**1**) with three different pyridine-containing coformers 4,4′-azopyridine (**2**), 4,4′-bipyridyl (**3**), and *trans*-1,2-bis(4-pyridyl)ethylene (**4**) and compare the photoreactivity of the resulting cocrystals with aliphatic amine salts of 4,4′-bipiperidine (**5**), morpholine (**6**) diethylamine (**7**), and *n*-butylamine (**8**) (Scheme 2). To date no X-ray structure information has been reported for PCDA or related photoactive surfactant-like molecules despite its commercial importance and hence no structure–reactivity relationship has been elucidated.

## Results and discussion

**Fig. 1:**
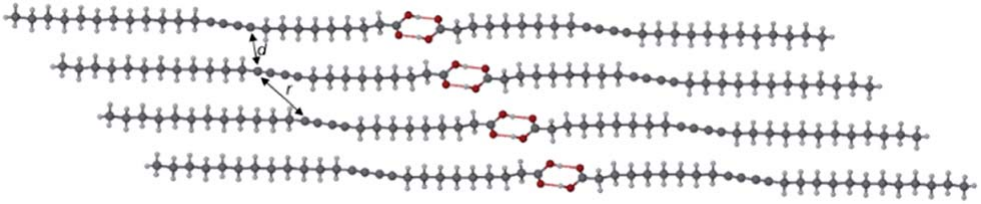
The X-ray structure of **1** showing the carboxylic acid dimer (O⋯O distance of 2.6581(9) Å) and the *anti*-conformation of the diacetylene substituents. The C1–C4′ distance and intermolecular repeat distance are denoted by *d* and *r*, respectively.

The X-ray crystal structure of PCDA is unknown and the preparation of single crystals of such surfactant-like compounds is generally regarded as difficult. However, preliminary powder X-ray diffraction (PXRD) analysis indicated that compound **1** is crystalline with a lamellar structure (ESI Fig. S1†). Slow evaporation of an acetone solution of PCDA gave high a quality sample suitable for analysis at the I19 beamline at the Diamond Light Source, Oxfordshire. Some decay of photosensitive **1** in the high-intensity synchrotron X-ray beam was noted, however, a structure determination was successfully carried out. The X-ray structure of **1** revealed a centrosymmetric structure (*P*1̄ space group) based on the well-known OH⋯O *R*_2_^2^(8) ring hydrogen-bonded carboxylic acid dimer synthon^55,56^ (Fig. 1). The structure is based on a head-to-head bilayer arrangement of molecules and the aliphatic substituents at either end of the dialkyne unit adopt an *anti*-conformation. The crystallographic *c*-axis of 46.647(3) Å is long, which is also apparent in the PXRD pattern, which shows a lamellar progression of low-angle peaks from 1.9–17.1° 2*θ*, all correlating to reflections in the (00*l*) plane (Fig. S1†).^57^ The structure conforms to the topochemical postulate for photoreactivity with a translational repeat distance of ≤4.9 Å, at 4.574(1) Å, along with a tilt angle of 44.7° that very close to the desired value of 45°. The inter-alkyne C1–C4′ distance between adjacent molecules of **1** is 3.712(1) Å, which is close to the upper limit of the topochemical postulate for alkyne reactivity (a maximum distance of 3.8 Å).^3^ These values are consistent with the limited observed solid state photoreactivity of **1** which, while it gradually turns visually blue upon exposure to UV and X-ray, the actual photoconversion as monitored by CP-MAS ^13^C NMR spectroscopy displays no alkene peaks, implying less than 1% even upon prolonged exposure (Fig. S2–S5†). Therefore, the question is raised as to whether the topochemistry of **1** can be engineered to provide an altered and tunable radiation response of use in radiation dosimetry by incorporating **1** into a cocrystal or a salt. The lithium salt of PCDA has been reported in this context in the patent literature,^58^ however, no X-ray crystal structure has been obtained to understand the photoreactivity of the salt. As a result, organic coformers potentially offer a structural insight to photoreactivity, along with improved versatility and more facile processing because of enhanced solubility.

### PCDA cocrystals

**Fig. 2:**
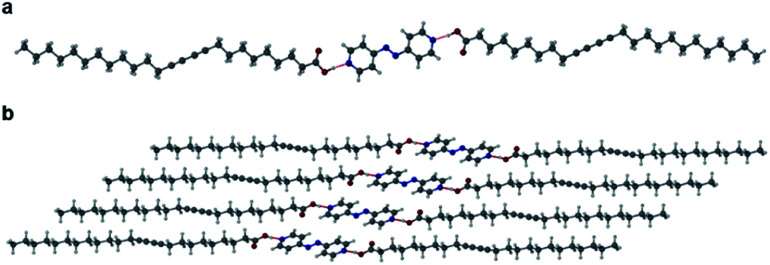
(a) The X-ray structure of **12·2** showing the OH⋯N hydrogen bond (O⋯N distance of 2.677(4)) with the diacetylene substituents in an *anti*-conformation. (b) The packing diagram of **12·2** in the (100) crystallographic plane.

Grinding PCDA (**1**) and 4,4′-azopyridine (**2**) in a Retsch MM 200 mixer mill for 1 hour in a 2 : 1 ratio, respectively (which reflects the single hydrogen bond donor group of **1** and the two hydrogen bond acceptor groups of **2**), gave a powder of cocrystal **12·2**, which was characterised by PXRD and used for seeding the cocrystallisation of **1** and **2** in acetone. No other stoichiometries were attempted. After the evaporation of solvent at room temperature for one week, plate-shaped crystals of **12·2** formed and were analysed by single crystal X-ray diffraction (SC-XRD). The structure of **12·2** reveals a 2 : 1 stoichiometry with the diacetylene substituents in *anti*-conformation, analogous to the structure of **1**, with OH⋯N hydrogen bonds from the carboxylic acid protons of **1** to the pyridyl nitrogen atoms of **2** (Fig. 2a). The O⋯N distance of 2.677(4) Å is consistent with a strong, carboxylic acid OH⋯pyridyl hydrogen bond. The carboxylic acid proton was located experimentally in the X-ray structure and is situated on the oxygen atom of the carboxylic acid, ruling out the possibility of salt formation. The unit cell of **12·2** has a shorter crystallographic *c*-axis of 39.920(2) Å compared to **1** itself (by a considerable 6.87 Å) implying a more slanted orientation of the lamellar structure (Fig. 2b). Compared to **1**, the cocrystal **12·2** also has a significantly shorter inter-alkyne C1–C4′ distance of 3.633(1) Å, however, the tilt angle of **1** in the cocrystal is greater than the optimum value at 48.4°, along with a translational repeat distance outside of the desired range for topochemical reactivity at 5.354(1) Å.

Along with SC-XRD, **12·2** was characterised by Differential Scanning Calorimetry (DSC) which displayed a melting onset temperature of 57 °C (Fig. S6†), which is lower than the melting temperatures of the individual components (62 °C for **1** (Fig. S7†) and 96 °C for **2** (ref. 59)) implying relatively weak interactions. The Fourier-transform infrared (FTIR) spectrum displays a hydrogen-bonded carbonyl stretching band at 1695 cm^−1^, compared to 1692 cm^−1^ in pure **1** implying slightly weaker hydrogen bonding (Fig. S8†). The cocrystal displays significant anisotropic thermal expansion along the *c*-axis which increases from 39.33 Å to 40.99 Å between 120 and 273 K. The differences in the unit cell made the calculated and experimental PXRD data difficult to compare, although it is clear that the single crystal studied is representative of the bulk material (Fig. S9†).

**Fig. 3:**
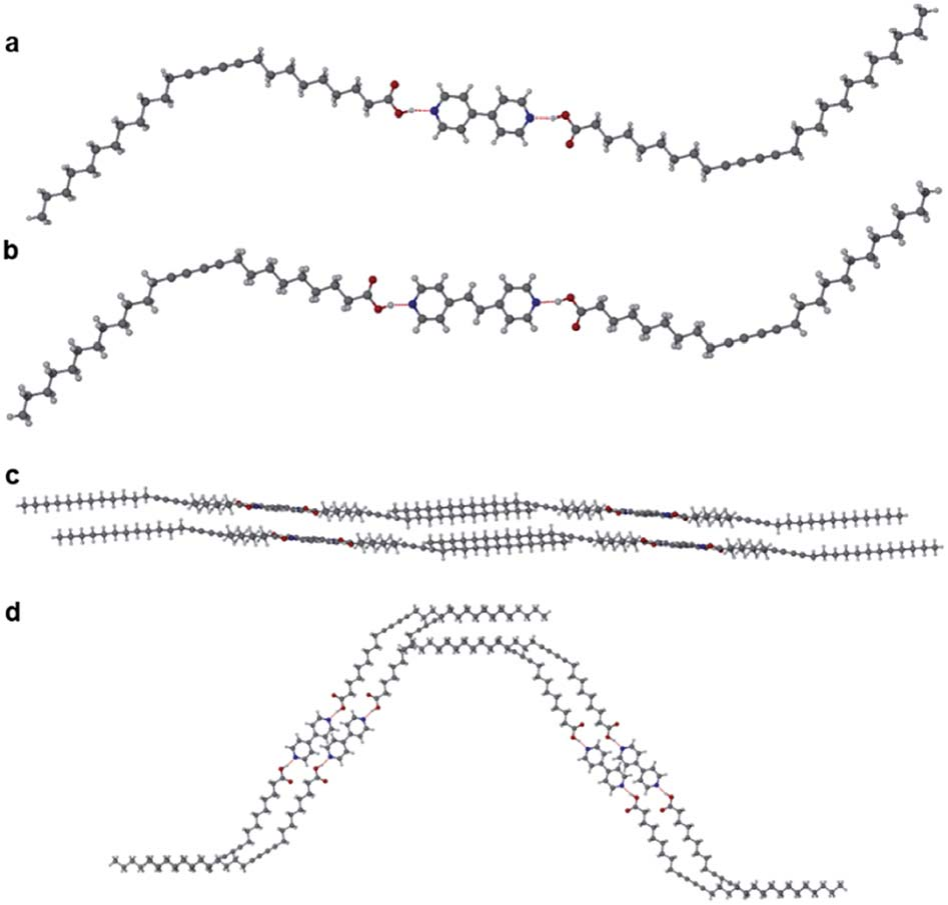
(a) The X-ray structure of **12·3** with components joined by an OH⋯N hydrogen bond (O⋯N distance of 2.6448(9) Å) showing the *syn*-conformation of the dialkyne substituents. (b) The X-ray structure of **12·4** with OH⋯N hydrogen bonds (O⋯N distance of 2.6579(17) Å) and the diacetylene substituents in a *syn*-conformation (disorder omitted). Packing diagram of **12·3** in the (c) (010) and (d) (001) crystallographic planes.

Two further cocrystals **12·3** and **12·4** were synthesised from 4,4′-bipyridyl and *trans*-1,2-bis(4-pyridyl)ethylene, respectively, by grinding the coformers with PCDA in a 2 : 1 ratio for 45 minutes in a mixer mill, to yield the cocrystal in powder form. Samples were characterised by PXRD and then used in seeded crystallisations in acetone. These experiments gave plates of **12·3** and **12·4** after the evaporation of solvent at room temperature for one week. A single crystal of **12·3** was analysed at the I19 beamline at the Diamond Light Source at 100 K, while crystals of **12·4** were analysed on a Bruker D8 Venture diffractometer at 120 K. The two materials are isostructural and crystallise in the monoclinic space group *P*2_1_/*c*. The X-ray structures of cocrystals **12·3** and **12·4** consist of hydrogen bonds between the carboxylic acid hydrogen atom of **1** and the pyridyl nitrogen atom of the coformer at an O⋯N distance of 2.652(4) Å in **12·3** (Fig. 3a) and 2.6579(17) Å in **12·4** (Fig. 3b). Interestingly the dialkyne moieties in both structures adopt a *syn*-conformation, in contrast to the *anti*-conformation in **1** and **12·2** indicating that subtle modification of conformer can have a significant effect on crystal packing mode. The X-ray structures of **12·3** and **12·4** and their packing diagrams are shown in Fig. 3c and d. The ethylene bond of **12·4** is disordered over two positions. The C1–C4′ inter-alkyne distances between adjacent PCDA molecules in both cocrystals are both within the topochemical postulate at distances of 3.730(1) Å and 3.726(2) Å, respectively, although they are longer than those found in **1** and **12·2**, because of the *syn* conformation of the dialkyne fragments. However, the tilt angle of PCDA in the cocrystals and the translational repeat distance for **12·3** is 47.4° and 5.442(1) Å, respectively, with similar values for **12·4** with a tilt angle of **1** in the cocrystal at 47.3° and a repeat distance of 5.449(2) Å. Therefore, neither of the isostructural cocrystals are expected to be photoreactive, as only one out of three of the parameters are within the topochemical postulate. The *syn* conformation of the dialkyne substituents allows an interdigitated, bilayer packing arrangement which translates to the much longer crystallographic *c* axes which encompass four folded molecules in the cocrystals of **3** and **4** as opposed to two extended molecules in **12·2**.

The DSC thermogram of **12·3** displays a melt onset endotherm of 73 °C (Fig. S10†) (compared to the coformer melt temperatures for **1** and **3** of 62 °C and 114 °C,^60^ respectively), while **12·4** exhibits a melting onset temperature of 72 °C (Fig. S11†), compared to 150 °C for **4**.^61^ The similar melting temperatures for the two cocrystals are expected due to the isostructural nature of these materials. The FTIR spectra for these cocrystals display a hydrogen-bonded carbonyl stretch at 1683 cm^−1^ and 1688 cm^−1^ respectively, compared to 1692 cm^−1^ in pure **1**, implying slightly stronger hydrogen bonding (Fig. S12 and S13†). In a similar way to **12·2**, cocrystals **12·3** and **12·4** show considerable anisotropic thermal expansion on warming (see Table S1†). This makes the calculated PXRD patterns appear somewhat different to the room temperature experimental patterns (Fig. S14 and S15†).

### PCDA salts

**Fig. 4:**
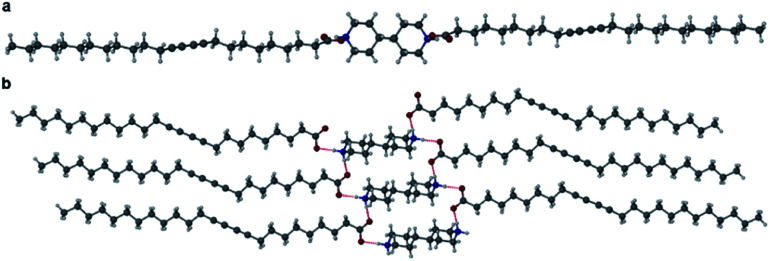
The X-ray structure of the salt cocrystal **12·5** in the (a) (100) and (b) (001) crystallographic planes.

Cocrystals of PCDA with bifunctional coformers **2–4** appear to give structures that are unlikely to be photoreactive based on their topochemical metrics. As a result, we examined both mono- and bifunctional coformers with higher basicity intended to deprotonate the PCDA acid functionality and hence alter the hydrogen bonding pattern and change the consequent stacking of the PCDA units. Salt formation was undertaken with a bifunctional diamine (**5**), a cyclic amine (**6**), a linear secondary amine (**7**), and a linear terminal amine (**8**). PCDA and compounds **5–8** were mechanochemically ground in a mixer mill to give a range of new salt materials as indicated by FTIR analysis. The carboxylate asymmetric carbonyl stretching modes proved to be at lower wavenumbers than in the free acid (**1**, 1692 cm^−1^) with a carbonyl stretch at 1653 cm^−1^ in **12·5** (Fig. S16†) and **1·6** (Fig. S17†), 1627 cm^−1^ in **12·7** (Fig. S18†), and 1649 cm^−1^ in **1·8** (Fig. S19†), suggesting stronger hydrogen bonding in the salts than the cocrystals and a delocalised carboxylate anion structure. The X-ray structure of **12·5** reveals a salt with two anions of **1** and a dication of double protonated **5** in a 2 : 1 stoichiometry, respectively, consisting of NH⋯O hydrogen bonds from the amine hydrogen atom of **5** and the oxygen atom of **1**, at an N⋯O distance of 2.717(1) Å (Fig. 4). The salt **12·5** crystallises with the same symmetry as **1** and **12·2** in the space group *P*1̄, with the crystallographic *c*-axis at the shortest observed so far at 23.0041(15) Å. The C1–C4′ inter-alkyne distance between adjacent molecules of **1** is 3.760(2) Å, which is within the topochemical postulate for the reactivity of diacetylenes (≤3.8 Å), however, the tilt angle of **1** in the salt cocrystal is below the desired value (45°) at 24.1°, and the translational repeat distance of 5.577(2) Å is outside the maximum distance for this parameter (≤4.9 Å) again suggesting limited photoreactivity.

**Fig. 5:**
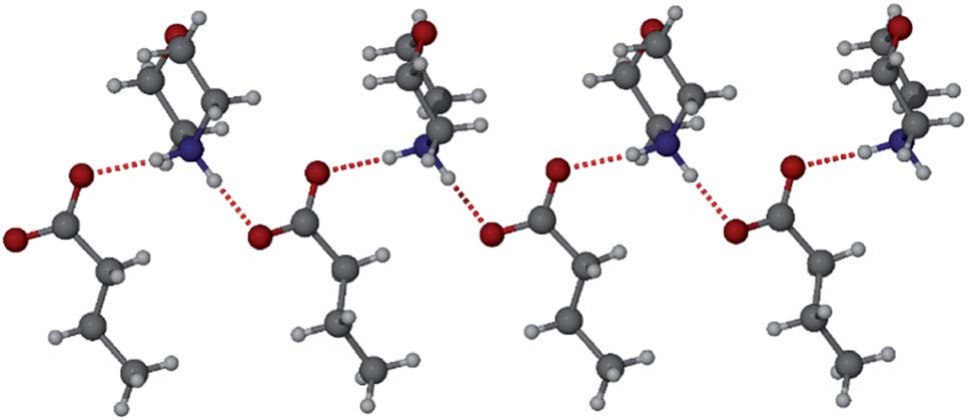
The X-ray structure of BuA·**6** showing two different hydrogen bonding interactions.

The morpholinium salt **1·6** was crystallised by the slow evaporation of acetone at room temperature, however, due to poor crystal quality after repeated crystallisation attempts, no SC-XRD analysis of **1·6** could be undertaken. To model the interactions between the two components, the synthesis of the butanoic acid (BuA) salt of **6** was attempted. Large single crystals of BuA·**6** formed from equimolar amounts of reagents in a sealed flask allowed to stand overnight. The X-ray structure reveals a salt with a butanoate anion and protonated morpholinium cation (Fig. 5). The structure involves two unique NH⋯O hydrogen bonding interactions with N⋯O distances of 2.673(1) Å and 2.732(1) Å. Based on the similar p*K*_a_ of **1** and butanoic acid it is possible that **1·6** is also a salt with similar head-group structure, although the relevance of this model system to the PCDA analogue is otherwise limited.

**Fig. 6:**
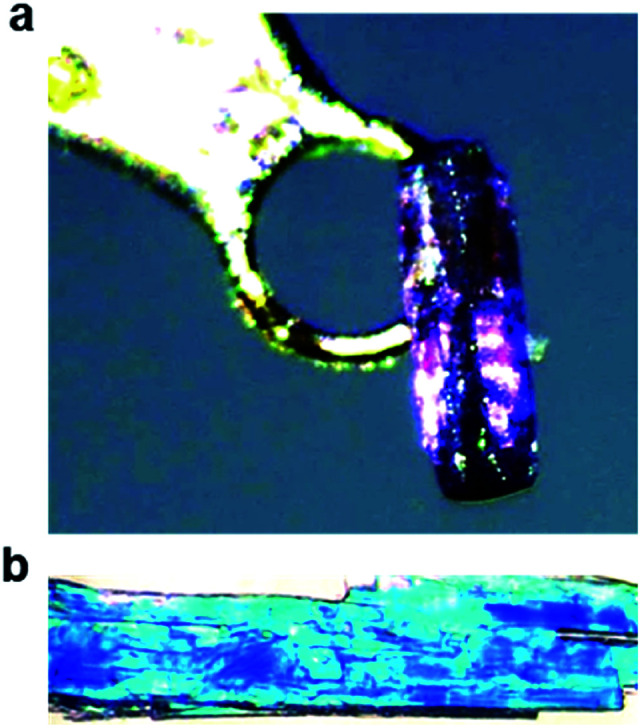
Photographs of (a) the purple crystal of **12·7** and (b) the blue crystals of **1·8**, taken before X-ray irradiation.

**Fig. 7:**
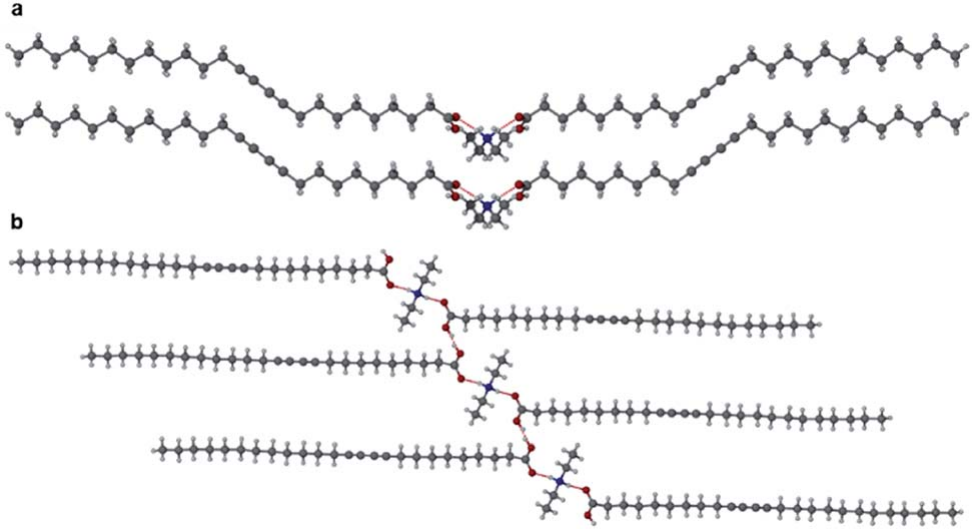
The X-ray structure of **12·7** in the (a) (100) and (b) (010) crystallographic planes.

**Fig. 8:**
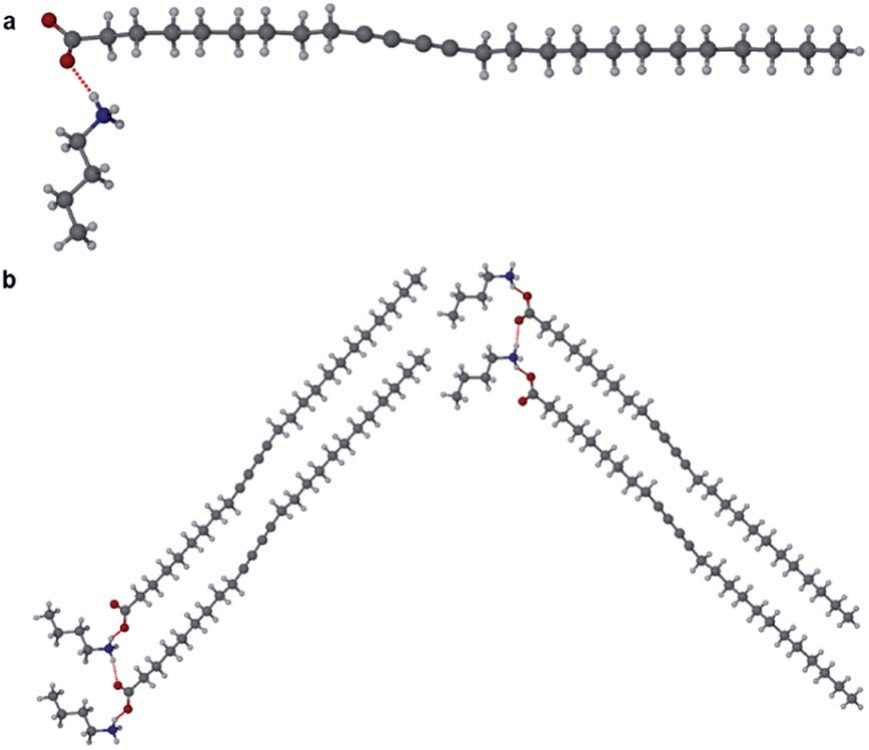
(a) The X-ray structure of **1·8** (b) in the (100) crystallographic plane.

Salts of PCDA with diethylamine (**7**) and *n*-butylamine (**8**) crystallised by slow evaporation of acetone solutions at room temperature. Surprisingly the crystals are highly coloured purple and blue, respectively, consistent with facile photopolymerisation (Fig. 6). However, the X-ray structure determinations reveal salts of unpolymerised PCDA and hence the colouration is likely to be a surface effect. Indeed, cutting a single crystal in half revealed a colourless inner core. The structure of the diethylammonium salt proved to be a salt cocrystal that also includes a neutral molecule of **1** (Fig. 7), with formula **12·7**. The butylammonium compound is a 1 : 1 salt of formula **1·8**. The structure adopts a stacked bilayer arrangement (Fig. 8). In **12·7,** hydrogen bonding occurs from the ammonium NH hydrogen atoms to the carbonyl oxygen of **1**, with an N⋯O distance of 2.737(1) Å. The carboxylic acid group of the neutral PCDA hydrogen bonds to the carboxylate functionality on the PCDA anion with a very short O⋯O distance of 2.444(1) Å (the additional hydrogen atom present between PCDA and the PCDA anion is disordered). In the 1 : 1 salt **1·8**, there are three different hydrogen bond interactions form from the NH_3_^+^ cation to the carboxylate oxygen atoms of the PCDA anion, with NH⋯O distances of 2.671(1) Å, 2.725(1) Å, and 2.784(1) Å. The **12·7** structure also has a large *c*-axis of 57.520(4) Å, which is the longest *c*-axis of all the structures studied reflecting the linear, parallel arrangement of the PCDA components. Salts **12·7** and **1·8** have similar C1–C4′ inter-alkyne distances of 3.776(2) Å and 3.779(1) Å, respectively, with tilt angles of 41.9° and 43.7°, and translational repeat distances of 4.644(3) Å and 4.593(1) Å. For these two salts, all three values are well within the optimum values of the topochemical postulate, and they are therefore are expected to show significant photoreactivity, consistent with the spontaneous surface colouration of the crystals.

DSC analyses of the PCDA salts of **5–8** reveal melt onset endotherms of 107 °C for **12·5** (Fig. S20†) (compared to 62 °C and 170 °C (ref. 62) for the parent components **1** and **5**, respectively). This relatively high value likely reflects the fact that proton transfer has occurred as well as the higher melting point of the bipiperidine coformer. The morpholinium salt **1·6** has a low melting onset of 44 °C (Fig. S21†) consistent with the fact that morpholine is a liquid at room temperature (it boils at 128 °C).^63^ In the same way as **1·6**, the DSC thermogram of **12·7** exhibits a melt onset endotherm of 46 °C (Fig. S22†), in comparison to the boiling temperature of 55 °C for **7**,^64^ while **1·8** displays a melt onset endotherm at 57 °C (Fig. S23†), with the salt former **8** boiling at 77 °C.^65^

### Cocrystal and salt response to UV and X-rays

**Fig. 9:**
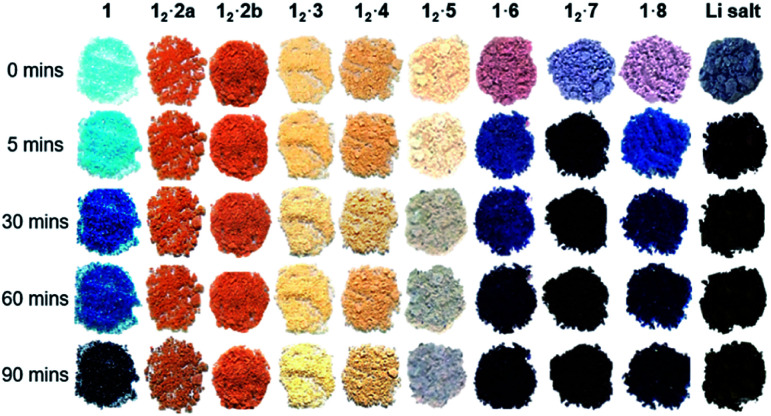
The coformer **1** and cocrystals **12·2**, **12·3** and **12·4**, and salts **12·5**, **1·6**, **12·7**, **1·8** and the lithium salt (Li salt) before and after UV irradiation at 254 nm for different durations. The **12·2a** sample was irradiated with UV light at 365 nm while 254 nm radiation was used for the **12·2b** sample. The initial colour changes to UV radiation show that only **1** and the salt samples visibly change colour with prolonged irradiation, with **12·7** being the most radiation sensitive to the naked eye and experimentally.

**Fig. 10:**
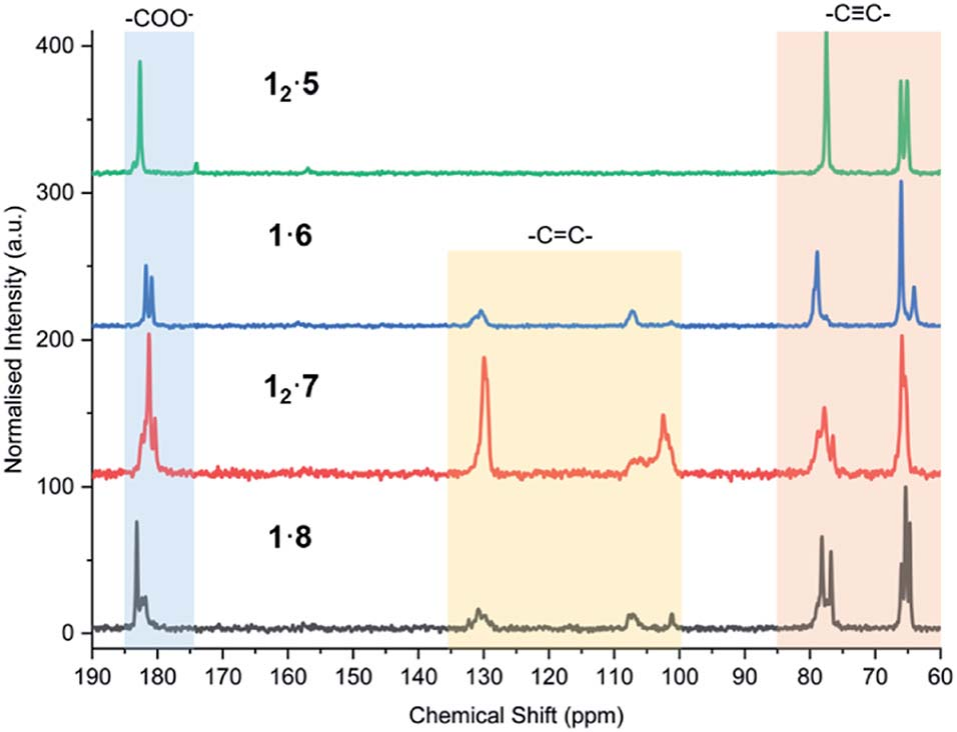
CP-MAS ^13^C NMR spectra of **12·5**, **1·6**, **12·7**, and **1·8** after 7 days of UV irradiation at 254 nm, highlighting the alkyne carbons (60–85 ppm), alkene carbons of the photoreactive salts (100–135 ppm), and carboxylate environment of each salt (175–185 ppm).

The powder of each cocrystal and salt was placed on filter paper in a dark box and exposed to a 6-Watt handheld UV light at 254 nm for varying durations (Fig. 9). It is known that the azobenzene coformer **2** itself undergoes photoisomerisation to the *cis* form when irradiated at 365 nm (ref. 66) and so **12·2** was also irradiated at this wavelength in order to probe photoresponse of the coformer component within the cocrystal. While PCDA powder itself gradually darkens from a white to deep blue upon irradiation, all of the cocrystals with coformers **2–4** do not change colour despite the close proximity of the dialkyne functionalities, which are within the distance specified by the topochemical postulate. However, the tilt angles of **1** in the cocrystals, and the translational repeat distances of the cocrystals are outside of the desired values. The irradiated cocrystals were analysed by PXRD, solid-state CP-MAS ^13^C NMR spectroscopy, and FTIR spectroscopy. This data confirmed that the cocrystal samples do not undergo photopolymerisation after irradiation (Fig. S24–S32†). However, after one hour and one day, **12·2** and **12·4** after one day, display additional peaks in the PXRD patterns and solid-state NMR spectra that are attributable to **1**. This suggests that irradiation decomposes the cocrystals to liberate free **1**, particularly in the case of the azobenzene complex **12·2**. This behaviour is attributed to the photoisomerisation of coformer **2** resulting in degradation of the cocrystal. The PXRD pattern and solid-state NMR spectra also imply a less-crystalline material at one hour in **12·2** and show the presence of crystalline **1** after one day of irradiation. In addition, the solid-state NMR spectroscopic data revealed that even the deep blue colour of the irradiated sample of PCDA is a surface effect and the material undergoes <1% photopolymerization, implying that the radiation is not penetrating the bulk of the sample. The bipiperidinium salt **12·5** is also not photoresponsive (Fig. S33 and S34†), consistent with the unfavourable translational repeat distance observed in the X-ray structure. From these results, bifunctional salt/coformers in general seem to give rise to a slightly offset packing mode that consistently results in an unfavourable repeat distance and tilt angle and hence essentially no photosensitivity. In contrast, the salts of monofunctional ammonium cations are all highly photoactive. Significant visual colour change occurs after just five minutes of irradiation for the salts **1·6**, **12·7** and **1·8** (Fig. 9). Signals assigned to photopolymerised material are clearly visible by CP-MAS ^13^C NMR spectroscopy (Fig. 10 and S35–S37†). Salt **12·7** shows the greatest sensitivity towards UV radiation by CP-MAS ^13^C NMR spectroscopy with the most significant change occurring in the alkene region (100–140 ppm) of the spectrum corresponding to the ene–yne photopolymer functionality (Fig. 10). However, even in these systems the conversion is slow, and the sharpness of the NMR resonances imply a relatively low degree of oligomerisation. This kind of slow reactivity reflects the solid-state nature of the process resulting in poor radiation penetration into the bulk of the sample. However, this gradual response is desirable in dosimetry applications making these materials of considerable interest. The significant photoreactivity of **12·7** and **1·8** is consistent with the crystal packing revealed by their structures, which both show parameters within the range specified by the topochemical postulate. While structural data is not available for the morpholinium salt, it seems likely that this too is within the topochemical postulate range. The topochemical parameters for each compound are summarised in Table 1.

**Table 1:** Topochemical parameters for PCDA cocrystals and salts structurally characterised in this work. The tilt angle (*θ*) is calculated from the orientation of **1** in the cocrystal and salt cocrystal samples

Compound	*d*/Å	*r*/Å	*θ*/°
**1**	3.712(1)	4.574(1)	44.7
**12·2**	3.633(1)	5.354(1)	48.4
**12·3**	3.730(1)	5.442(1)	47.4
**12·4**	3.726(2)	5.449(2)	47.3
**12·5**	3.760(2)	5.577(2)	24.1
**12·7**	3.776(2)	4.644(3)	41.9
**1·8**	3.779(1)	4.593(1)	43.7

**Fig. 11:**
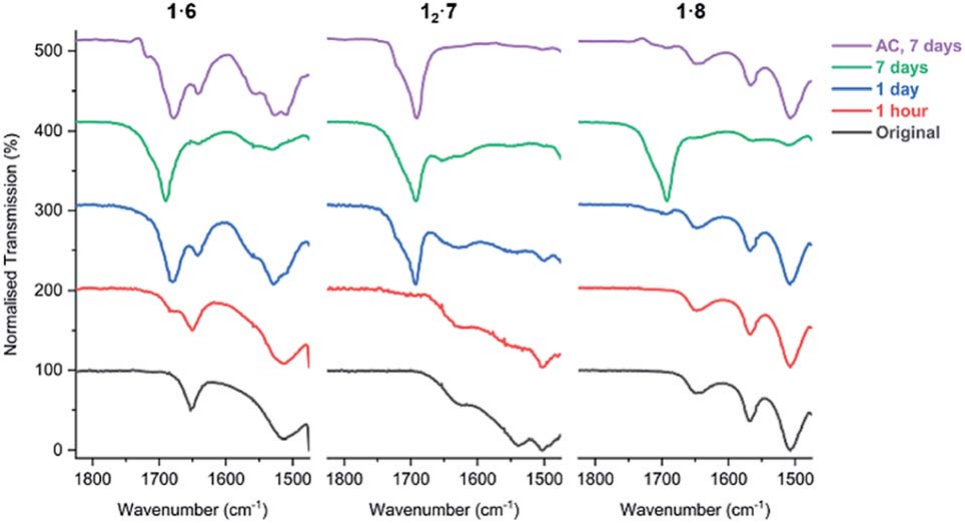
The FTIR spectra of salts **1·6**, **12·7**, and **1·8** in the range of 1475–1825 cm^−1^ showing the carboxylate environment of the salts with volatile amines as the samples are irradiated with UV light (254 nm), and exposure to ambient conditions (AC) for seven days.

FTIR analysis of the salt cocrystals after irradiation shows that salts **1·6**, **12·7**, and **1·8** begin to lose their volatile coformers after prolonged UV exposure and revert to free carboxylic acids. This is evidenced by the decrease in intensity of the carboxylate asymmetric stretch band *ν*_asymm_(CO_2_) of the salt (1653 cm^−1^ in **1·6**, 1627 cm^−1^ in **12·7**, and 1649 cm^−1^ in **1·8**) and the emergence of a free acid peak at 1692 cm^−1^ close to the value of PCDA as the sample is irradiated (Fig. 11). The effect is highly pronounced for the morpholinium salt **1·6** which reverts to free acid after just one hour while **7** and **8** begin to separate from their respective salts after one day of irradiation. The resulting carboxylic acid is a mixture of free PCDA and photopolymer. These findings are also supported by PXRD analysis of the irradiated salts (Fig. S38–S40†). Interestingly, given the very limited photoreactivity of PCDA itself, salt formation followed by removal of the amine in this way gives an interesting route to the free acid photopolymer and hence transient amine complexation effectively catalyses the photopolymerisation reaction of PCDA itself. The salts were also exposed to ambient conditions for seven days to investigate whether the amine is lost from the salts without irradiation. The FTIR of salts **1·6** and **1·8** display changes consistent with UV irradiation for one day, while salt **12·7** displays total loss of amine from the salt under ambient conditions after seven days (Fig. 11).

**Fig. 12:**
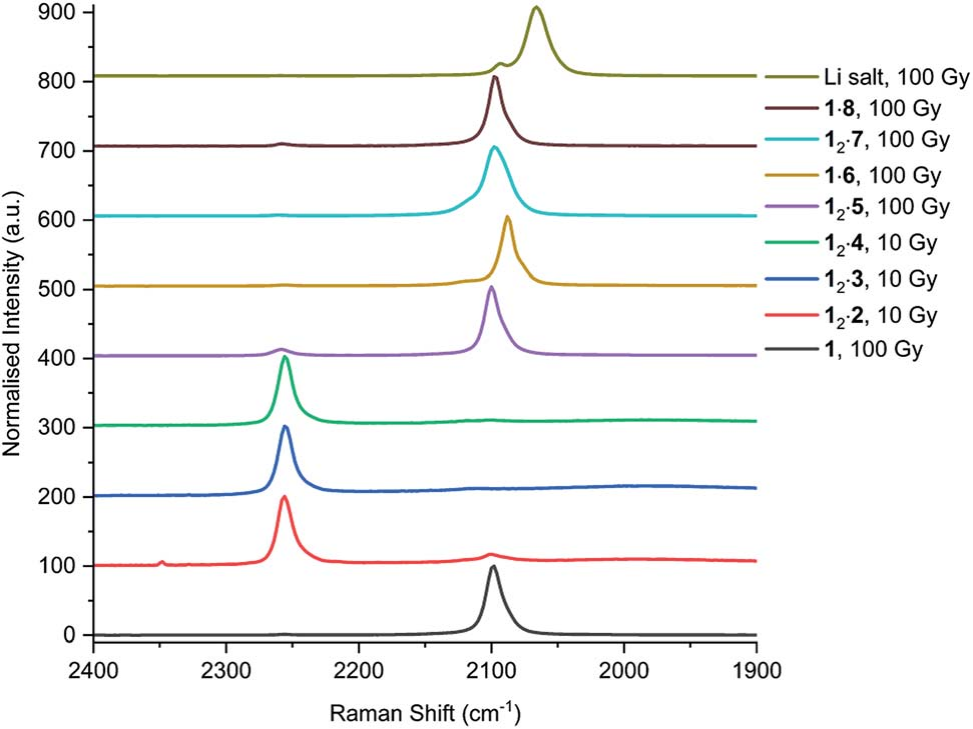
Raman spectra of **1**, and cocrystal and salts after X-ray irradiation. The peaks at approx. 2260 cm^−1^ correlate to the alkyne band of monomeric **1**, while the internal alkyne of the photopolymer is displayed at approx. 2100 cm^−1^.

**Fig. 13:**
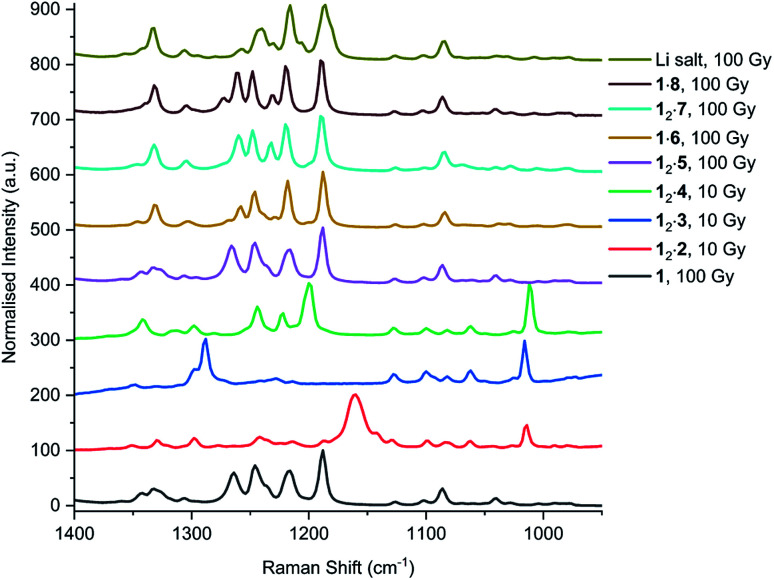
The methylene wagging vibrations of **1** and each cocrystal and salt after X-ray irradiation, analysed by Raman spectroscopy.

In addition to UV irradiation, the effect of X-ray on PCDA and its derivatives was also analysed. Free PCDA (**1**) was irradiated with 100 Gy of X-ray radiation and analysed by Raman spectroscopy (Fig. 12). This revealed a clear ene–yne polymer alkyne band at 2098.8 cm^−1^ with a small residual dialkyne band at 2253.3 cm^−1^ (Fig. S41†). The enhanced appearance of the 2098.8 cm^−1^ band despite the very limited photoreactivity of free **1** is a reflection of a pre-resonance Raman effect since the excitation wavelength of the laser 785 nm overlaps with the absorption band of the photopolymer, resulting in significant enhancement of the chromophore Raman bands. This is consistent with the visual observation of some blue colouration despite the ^13^C CP MAS-NMR data that indicate a very low degree of bulk conversion (Fig. S4†). In contrast, when all three cocrystals with coformers **2–4** were irradiated with 10 Gy of X-ray radiation they showed very little photoreactivity, as evidenced by the low intensity peaks in the conjugated ene–yne region (approx. 2100 cm^−1^)^67^ that exists both before and after irradiation (Fig. S42–S44†). Cocrystal **12·2** has a small ene–yne band present at 2100.4 cm^−1^ compared to the other two cocrystals likely arising from small amounts of **1** photopolymer present as a contaminant in the starting PCDA. Similarly to the cocrystals, salt **12·5** displays a band at 2258.4 cm^−1^ assigned to unreacted dialkyne even after 100 Gy of X-ray irradiation which further reinforces that the salt is photostable (Fig. S45†). The small ene–yne photopolymer band at 2100.3 cm^−1^ is likely to arise from small amounts of photopolymerised PCDA impurities. On the other hand, salt **1·6** shows impressive sensitivity to X-ray radiation as indicated by the presence of the significant ene–yne band at 2088.1 cm^−1^ (Fig. S46†). This band is significantly red-shifted compared to photopolymerised PCDA, indicating a more planar, conjugated conformation of the chromophore. This is in contrast to **1** alone which exhibits torsional strain on the π-bonds of the chromophore when irradiated.^68^ Salt **1·6** also shows significantly more visual colour change upon irradiation compared to **1** alone. It is likely that the increased hydrogen bonding in the salt brings the monomers of **1** in a closer spatial arrangement and hence makes it more photosensitive. After 100 Gy of X-ray irradiation, salt **12·7** also displays a prominent photopolymer alkyne band at 2097.7 cm^−1^ with minimal residual dialkyne signal (Fig. S4†). The pre-resonance Raman effect is very evident in **12·7** and can be seen in the exaggeration of the ene–yne band in Fig. 12 relative to the dialkyne band in the region of 2250 cm^−1^. Solid-state NMR results indicate about 53% polymerisation (Fig. S47†), however the Raman signal for the colourless monomer is almost invisible. Interestingly in the Raman spectrum of **12·7**, the breadth of the ene–yne band, and the presence of an additional alkene peak at 1500 cm^−1^ at slightly higher wavenumber than the typical major 1445 cm^−1^ alkene band indicates multiple conformations of the polymerised material, and implies some structural differences in the resulting chromophore suggesting that multiple conformations of the polymerised salt exist. For **1·8**, Raman analysis of the 100 Gy X-ray irradiated sample shows that the salt gradually photopolymerises and has a similar radiation sensitivity as **1** alone, with an ene–yne band at 2098.1 cm^−1^ (Fig. S48†). Additionally, for the photosensitive salts, the C–H wagging progressions arising between 1300 cm^−1^ and 1150 cm^−1^ from the polymer side chains of **1** in the salts change with irradiation to suggest a changed conformational structure when compared to the lithium salt (Fig. 13). The change of the side chain conformation is due to difference in phase angles of coupled oscillations between methylene groups. These differences in C–H wagging progressions can be used as an additional conformational tool for detecting the presence of a PCDA polymer. Close examination of the differences in frequency within the wagging mode progressions may also indicate stresses on the side chains due to their close approach to each other as the polymer is formed. Interestingly, the positions of the ene–yne alkyne bands in the irradiated diethylammonium and butylammonium salts of around 2100 cm^−1^ contrast sharply with the value of 2066.3 cm^−1^ obtained for commercial lithium PCDA. This significantly red-shifted value implies a much more planar ‘ordered’ chromophore in the lithium salt and hence while the commercial material exists in an ordered ‘blue state’ the use of the organic salt-formers give a less ordered ‘red state’ photopolymer. The value of 2088 cm^−1^ for the morpholinium salt is somewhere in between and implies that the polymer ordering and hence, potentially, colour may be tunable.

In an attempt to assess the degree of polymerisation, the irradiated samples were analysed by two different mass spectrometry techniques, matrix-assisted laser desorption/ionisation (MALDI) and atmospheric solid analysis probe (ASAP). However, neither method was able to fully quantify the amount of polymer present in the samples nor the molecular weight distribution. MALDI did not generate any signals assignable to photopolymerised material. In contrast, the ASAP technique did show peaks assigned to PCDA monomer, dimer, and trimer, however, the distribution of these signals was the same before and after UV irradiation for one day for PCDA itself and even for **12·7** UV irradiated for 14 days. It is likely that higher molecular weight oligomers are present given the broadness of the CP-MAS NMR spectra, therefore, these are not volatile enough to be detected by mass spectrometry in this way. Similarly, the poor solubility of the polymerised materials made them unsuitable for gel permeation chromatography.

## Conclusion

Three PCDA cocrystals **12·2**, **12·3**, and **12·4** have been prepared which were engineered to exhibit the common carboxylic acid⋯pyridine supramolecular synthon. The lamellar structures of these surfactant-like compounds exhibit two very different packing modes with either a *syn* or *anti* conformation of the dialkyne substituents and display considerable thermal expansion in the *c*-axis direction upon warming. The cocrystal with the shortest dialkyne distance of 3.631(6) Å is **12·2**, which is shorter than in PCDA itself and all cocrystals have inter-alkyne distances within the topochemical postulate. However, they all exhibit very little response to UV and X-ray radiation, because the translational repeat distances are greater than the maximum value. Hence, cocrystallisation appears to significantly stabilise PCDA in the solid state. This observation is somewhat surprising in the light of the photoreactivity of related pyridyl-containing cocrystals of di- and trialkynes.^45,69^ In contrast, salts of monofunctional amines gave highly photoreactive materials. For example, the morpholinium salt **1·6** changes from pink-to-blue-to-black in under 10 minutes of UV irradiation. Similarly the salt cocrystal with diethylamine **12·7** and the butylammonium salt **1·8** are both highly radiation sensitive and undergo an impressive lilac-to-black colour change with under 5 minutes of UV irradiation for **12·7** and a pink-to-navy colour change for **1·8**, in powder form. Their X-ray crystal structures indicate that these salts adhere to the topochemical postulate. These radiation-sensitive salts are of considerable commercial interest for the development of radiochromic films and due to their different sensitivities to radiation, can be applied to different radiation dose ranges, and therapeutic areas. Preliminary indications suggest that they outperform the commercial lithium salt in terms of photosensitivity, while Raman spectroscopy shows that the photopolymers are relatively disordered with ene–yne bands in the range 2088–2100 cm^−1^, potentially allowing access to a range of colours. The novel feature of amine evaporation over time means that transient ammonium salt formation with a volatile amine effectively catalyses the solid-state photopolymerisation of the relatively unreactive PCDA.

## Experimental

### General

10,12-Pentacosadynoic acid (**1**) was supplied by Ashland LLC, with all other reagents and solvents purchased from standard commercial sources and used without further purification. IR spectra were measured with a PerkinElmer 100 FT-IR spectrometer with an uATR attachment. Raman spectra were collected on a PerkinElmer Ramanstation 400F with 5–10 accumulations of 10–60 second scans, using an excitation laser with a wavelength of 785 nm. Solid-state NMR spectra were recorded at 100.63 MHz using a Bruker Avance III HD spectrometer and a 4 mm magic-angle spinning probe. Spectra were obtained using cross-polarisation with a 20 s recycle delay with 7 ms contact time at ambient probe temperature (approx. 25 °C) at a sample spin rate of 10 kHz with 400 repetitions. Spectral referencing was with respect to an external sample of neat tetramethylsilane. Differential scanning calorimetry thermograms were recorded using a PerkinElmer 8500 calorimeter, calibrated using an indium standard, with samples accurately weighed (±0.01 mg) into standard aluminium pans.

Mass spectra by the ASAP technique were recorded using a LCT Premier XE mass spectrometer (Waters Ltd) heating approximately 1 mg of powder isothermally at 350 °C. Single crystal X-ray data for the cocrystals and salts **12·2**, **12·4**, **12·5**, BuA·**6**, and **12·7** were collected at 120.0(2) K on a Bruker D8 Venture diffractometer (Photon 100 CMOS detector, IuS-microsource focusing mirrors) equipped with Cryostream (Oxford Cryostreams) open-flow nitrogen cryostat and using Mo Kα and Cu Kα radiation with wavelengths of 0.71073 Å and 1.54178 Å, respectively. The single crystal data for **1·8** was collected at 120.0 K on an XCalibur Agilent, Sapphire3 diffractometer equipped with Cryostream 700 nitrogen cryostat and using Mo Kα radiation with a wavelength of 0.71073 Å. Single crystal data for **1** and **12·3** were collected at 100.0(2) K at I19 beamline (Dectris Pilatus 2M pixel-array photon-counting detector, undulator, graphite monochromator, *λ* = 0.6889 Å) at the Diamond Light Source, Oxfordshire. All structures were solved using direct methods and refined by full-matrix least squares on *F*^2^ for all data using SHELXL^50^ and OLEX2 software.^51^ All non-hydrogen atoms were refined with anisotropic displacement parameters. CH hydrogen atoms were placed in calculated positions, assigned an isotropic displacement factor that is a multiple of the parent carbon atom and allowed to ride. H atoms attached to oxygen atoms were located on the difference map when possible or placed in calculated positions. X-ray powder diffraction patterns were performed on glass slides, using a Bruker AXS D8 Advance diffractometer, with a Lynxeye Soller PSD Detector, using Cu Kα radiation at a wavelength of 1.5406 Å. CCDC deposition numbers 2000830–2000837.† The powdered cocrystal and salt samples were placed on filter paper in a dark box and exposed to a 6-Watt handheld UV light at 254 nm. Compound **12·2** was also irradiated at 365 nm. The powdered samples were mixed at regular intervals to ensure an even exposure of the bulk to irradiation.

### Synthesis of PCDA, cocrystals, and salts

#### 10,12-Pentacosadiynoic acid (**1**)

Analysis calc. of C_25_H_42_O_2_: C 80.16, H 11.30%, found: C 80.05, H 11.18%; FTIR (*ν*/cm^−1^): 2956, 2918, 2848, 1692, 1467, 1460, 1417, 1291, 1264, 932, 722. Colourless needle crystals of high quality were grown from the slow evaporation of acetone at room temperature for one week from a failed cocrystallisation experiment with pyrazine. Crystal data: *M* = 374.58 g mol^−1^, 0.12 × 0.04 × 0.01 mm^3^, triclinic, space group *P*1̄ (no. 2), *a* = 4.5738(3) Å, *b* = 5.3909(3) Å, *c* = 46.647(3) Å, *α* = 88.6499(15)°, *β* = 88.5073(14)°, *γ* = 81.4017(14)°, *V* = 1136.64(12) Å^3^, *Z* = 2, *D*_c_ = 1.094 g cm^−3^, *F*_000_ = 416.0, synchrotron radiation, *λ* = 0.6889 Å, *T* = 100 K, 2*θ*_max_ = 58.994°, 21 023 reflections collected, 6893 unique (*R*_int_ = 0.0574). Final GooF = 1.035, *R*_1_ = 0.0640, w*R*_2_ = 0.1772, *R* indices based on 6893 reflections with *I* ≥ 2*σ*(*I*) (refinement on *F*^2^), 249 parameters, 0 restraints, *μ* = 0.063 mm^−1^.

#### PCDA 4,4′-azopyridine cocrystal (**12·2**)

Cocrystal **12·2** was prepared by grinding **1** (0.15 g, 0.40 mmol) and **2** (0.037 g, 0.20 mmol) in a 2 : 1 ratio, respectively, in a Retsch MM 200 mixer mill for 1 hour at a frequency of 20 s^−1^ (yield 0.10 g, 0.22 mmol, 55%). The resulting powder was used as a seed (*ca.* 0.005 g, 0.011 mmol) for the cocrystallisation of **1** (0.025 g, 0.067 mmol) and **2** (0.006 g, 0.033 mmol) in acetone (2 mL). After brief sonication, the solution was left to crystallise by slow evaporation at room temperature, which yielded large colourless plate crystals. Analysis for C_30_H_46_N_2_O_2_ calc.: C 77.19, H 9.94, N 6.01%, found: C 77.19, H 9.93, N 5.32%; FTIR (*ν*/cm^−1^): 2936, 2919, 2850, 1695, 1598, 1470, 1410, 1253, 1214, 1184, 1011, 848, 719, 573. Crystal data: *M* = 466.69 g mol^−1^, 0.32 × 0.09 × 0.02 mm^3^, triclinic, space group *P*1̄ (no. 2), *a* = 5.3544(3) Å, *b* = 6.8239(4) Å, *c* = 39.920(2) Å, *α* = 87.742(4)°, *β* = 88.869(4)°, *γ* = 75.291(4)°, *V* = 1409.64(14) Å^3^, *Z* = 2, *D*_c_ = 1.100 g cm^−3^, *F*_000_ = 512.0, Cu Kα radiation, *λ* = 1.54178 Å, *T* = 120 K, 2*θ*_max_ = 137.98°, 33 397 reflections collected, 5160 unique (*R*_int_ = 0.1445). Final GooF = 1.090, *R*_1_ = 0.0994, w*R*_2_ = 0.1798, *R* indices based on 5160 reflections with *I* ≥ 2*σ*(*I*) (refinement on *F*^2^), 312 parameters, 0 restraints, *μ* = 0.522 mm^−1^.

#### PCDA 4,4′-bipyridyl cocrystal (**12·3**)

Cocrystal **12·3** was prepared by grinding **1** (0.15 g, 0.40 mmol) and **3** (0.032 g, 0.20 mmol) in a Retsch MM 200 mixer mill for 45 minutes at a frequency of 20 s^−1^ (yield = 0.091 g, 0.20 mmol, 50%). The resulting powder was used as a seed (*ca.* 0.005 g, 0.011 mmol) for the cocrystallisation of **1** (0.025 g, 0.067 mmol) and **3** (0.053 g, 0.034 mmol) in acetone (2 mL). After brief sonication, the solution was left to crystallise by slow evaporation at room temperature, which yielded small colourless plate crystals. Analysis calc. of C_30_H_46_N_2_O_2_: C 79.60, H 10.24, N 3.09%, found: C 79.24, H 10.20, N 3.12%; FTIR (*ν*/cm^−1^): 2919, 2850, 1683, 1600, 1471, 1408, 1365, 1325, 1287, 1253, 1212, 1187, 1071, 1000, 821, 718, 625. Crystal data: *M* = 452.68 g mol^−1^, 0.12 × 0.04 × 0.03 mm^3^, monoclinic, space group *P*2_1_/*c* (no. 14), *a* = 5.4415(2) Å, *b* = 8.9535(4) Å, *c* = 55.673(3) Å, *β* = 90.8823(10)°, *V* = 2712.1(2) Å^3^, *Z* = 4, *D*_c_ = 1.109 g cm^−3^, *F*_000_ = 996.0, synchrotron radiation, *λ* = 0.6889 Å, *T* = 100 K, 2*θ*_max_ = 57.994°, 47 696 reflections collected, 7923 unique (*R*_int_ = 0.0663). Final GooF = 1.069, *R*_1_ = 0.0529, w*R*_2_ = 0.1510, *R* indices based on 7923 reflections with *I* ≥ 2*σ*(*I*) (refinement on *F*^2^), 303 parameters, 0 restraints, *μ* = 0.064 mm^−1^.

#### PCDA *trans*-1,2-bis(4-pyridine)ethylene cocrystal (**12·4**)

Cocrystal **12·4** was prepared by grinding **1** (0.15 g, 0.40 mmol) and **4** (0.039 g, 0.21 mmol) in a Retsch MM 200 mixer mill for 45 minutes at a frequency of 20 s^−1^ (yield = 0.12 g, 0.26 mmol, 65%). The resulting powder was used as a seed (*ca.* 0.005 g, 0.011 mmol) for the cocrystallisation of **1** (0.025 g, 0.067 mmol) and **4** (0.0061 g, 0.033 mmol) in acetone (2 mL). After brief sonication, the solution was left to crystallise by slow evaporation at room temperature and yielded small colourless block crystals. Analysis calc. of C_31_H_47_NO_2_: C 79.95, H 10.17, N 3.01%, found: C 79.84, H 10.14, N 2.86%; FTIR (*ν*/cm^−1^): 2919, 2850, 1688, 1604, 1471, 1414, 1325, 1252, 1235, 1213, 1183, 1100, 974, 826, 718, 553. Crystal data: *M* = 465.69 g mol^−1^, 0.36 × 0.31 × 0.23 mm^3^, monoclinic, space group *P*2_1_/*c* (no. 14), *a* = 5.4494(3) Å, *b* = 8.9235(5) Å, *c* = 57.441(3) Å, *β* = 92.643(2)°, *V* = 2790.2(3) Å^3^, *Z* = 4, *D*_c_ = 1.109 g cm^−3^, *F*_000_ = 1024.0, Mo Kα radiation, *λ* = 0.71073 Å, *T* = 120 K, 2*θ*_max_ = 56.98°, 34 945 reflections collected, 6537 unique (*R*_int_ = 0.0635). Final GooF = 1.034, *R*_1_ = 0.0560, w*R*_2_ = 0.1073, *R* indices based on 6537 reflections with *I* ≥ 2*σ*(*I*) (refinement on *F*^2^), 321 parameters, 1 restraint, *μ* = 0.067 mm^−1^.

#### PCDA 4,4′-bipiperidine salt (**12·5**)

Salt **12·5** was prepared by grinding **1** (0.2 g, 0.53 mmol) and **5** (0.045 g, 0.27 mmol) in a 2 : 1 ratio in a Retsch MM 200 mixer mill for 90 minutes at a frequency of 20 s^−1^ (yield = 0.22 g, 0.40 mmol, 89%). The resulting powder (0.030 g) was combined with ethanol (2 mL) and briefly sonicated and left to crystallise by slow evaporation at room temperature. Colourless plate crystals formed after 2 weeks. Analysis calc. of C_60_H_104_N_2_O_4_: C 78.55, H 11.43, N 3.05%, found: C 77.94, H 11.36, N 2.83%; FTIR (*ν*/cm^−1^): 2917, 2849, 1692, 1644, 1527, 1467, 1418, 1305, 1266, 1231, 1099, 1011, 921, 868, 807, 719, 639. Crystal data: *M* = 917.45 g mol^−1^, 0.767 × 0.314 × 0.1 mm^3^, triclinic, space group *P*1̄ (no. 2), *a* = 5.5770(4) Å, *b* = 11.8339(8) Å, *c* = 23.0041(15) Å, *α* = 100.670(2)°, *β* = 96.096(2)°, *γ* = 103.007(2)°, *V* = 1436.25(17) Å^3^, *Z* = 1, *D*_c_ = 1.061 g cm^−3^, *F*_000_ = 510.0, Mo Kα radiation, *λ* = 0.71073 Å, *T* = 120 K, 2*θ*_max_ = 65°, 36 532 reflections collected, 10 400 unique (*R*_int_ = 0.0455). Final GooF = 1.019, *R*_1_ = 0.0497, w*R*_2_ = 0.1263, *R* indices based on 10 400 reflections with *I* ≥ 2*σ*(*I*) (refinement on *F*^2^), 312 parameters, 0 restraints, *μ* = 0.064 mm^−1^.

#### PCDA morpholine salt (**1·6**)

Salt **1·6** was prepared by grinding **1** (0.2 g, 0.53 mmol) and **6** (0.046 mL, 0.53 mmol) in a Retsch MM 200 mixer mill for 45 minutes at a frequency of 20 s^−1^ (yield = 0.24 g, 0.52 mmol, 98%). The resulting powder of **1·6** (0.030 g) was combined with acetone (3 mL) and briefly sonicated and left to crystallise by slow evaporation at room temperature. Colourless plate crystals formed after 2 weeks, however, crystallisations with and without seeding yielded poor-quality crystals that did not diffract sufficiently to allow single crystal structure determination. Analysis calc. of C_29_H_51_NO_3_: C 75.45, H 11.14, N 3.03%, found C 75.18, H 11.08, N 2.80%; FTIR (*ν*/cm^−1^): 2917, 2850, 1652, 1515, 1474, 1406, 1297, 1108, 879, 728, 616.

#### Morpholinium butanoate salt (BuA·**6**)

Salt **1·6** was prepared by combining butanoic acid (0.05 mL, 0.55 mmol) and **6** (0.047 mL, 0.55 mmol) to give a yellow oil and was left to precipitate slowly overnight in a sealed round-bottom flask to yield large colourless plate crystals (yield = 0.088 g, 0.5 mmol, 91%). Analysis calc. of C_8_H_17_NO_3_: C 54.84, H 9.78, N 7.99%, found: C 54.38, H 10.06, N 7.70%; FTIR (*ν*/cm^−1^): 2961, 2871, 1711, 1545, 1456, 1394, 1379, 1303, 1243, 1195, 1107, 1049, 997, 878, 829, 766, 615. Crystal data: *M* = 175.22 g mol^−1^, 0.44 × 0.25 × 0.21 mm^3^, monoclinic, space group *C*2/*c* (no. 15), *a* = 20.0926(14) Å, *b* = 8.0678(6) Å, *c* = 11.6061(8) Å, *β* = 97.064(3)°, *V* = 1867.1(2) Å^3^, *Z* = 8, *D*_c_ = 1.247 g cm^−3^, *F*_000_ = 768.0, Mo Kα radiation, *λ* = 0.71073 Å, *T* = 120 K, 2*θ*_max_ = 58.994°, 13 471 reflections collected, 2587 unique (*R*_int_ = 0.0313). Final GooF = 1.023, *R*_1_ = 0.0356, w*R*_2_ = 0.0931, *R* indices based on 2587 reflections with *I* ≥ 2*σ*(*I*) (refinement on *F*^2^), 177 parameters, 0 restraints, *μ* = 0.094 mm^−1^.

#### PCDA diethylamine salt cocrystal (**12·7**)

Salt cocrystal **12·7** was prepared by grinding **1** (0.2 g, 0.53 mmol) and **7** (0.055 mL, 0.53 mmol) in a Retsch MM 200 mixer mill for 45 minutes at a frequency of 20 s^−1^ (yield = 0.22 g, 0.5 mmol, 94%). The resulting powder of **12·7** (0.030 g) was combined with acetone (3 mL) and briefly sonicated and left to crystallise by slow evaporation at room temperature. Purple plate crystals formed after 2 weeks to reveal a 2 : 1 (PCDA : diethylamine) stoichiometry with the formula (C_25_H_42_O_2_)·(C_25_H_41_O_2_)^−^·(C_4_H_12_N^+^). Analysis calc. for **12·7**: C 78.87, H 11.64, N 1.70%, found: C 77.47, H 11.61, N 1.70%; FTIR (*ν*/cm^−1^): 2923, 2846, 1627, 1461, 1423, 1384, 1068, 1010, 955, 854, 811, 724, 592. Crystal data: *M* = 822.3 g mol^−1^, 0.5 × 0.12 × 0.1 mm^3^, monoclinic, space group *P*2/*n* (no. 13), *a* = 9.5968(6) Å, *b* = 4.6441(3) Å, *c* = 57.520(4) Å, *β* = 92.590(2)°,*V* = 2561.0(3) Å^3^, *Z* = 2, *D*_c_ = 1.066 g cm^−3^, *F*_000_ = 916.0, Mo Kα radiation, *λ* = 0.71073 Å, *T* = 120 K, 2*θ*_max_ = 55.996°, 30 938 reflections collected, 6051 unique (*R*_int_ = 0.0502). Final GooF = 1.049, *R*_1_ = 0.0561, w*R*_2_ = 0.1217, *R* indices based on 6051 reflections with *I* ≥ 2*σ*(*I*) (refinement on *F*^2^), 274 parameters, 0 restraints, *μ* = 0.065 mm^−1^.

#### PCDA *n*-butylamine salt (**1·8**)

Salt **1·8** was prepared by grinding **1** (0.2 g, 0.53 mmol) and **8** (0.053 mL, 0.53 mmol) in a Retsch MM 200 mixer mill for 45 minutes at a frequency of 20 s^−1^ (yield = 0.23 g, 0.5 mmol, 96%). The resulting powder of **1·8** (0.030 g) was combined with acetone (2 mL) and briefly sonicated. A powder seed (*ca.* 0.004 g, 0.0089 mmol) was added to the sample and left to crystallise by slow evaporation at room temperature. Blue block crystals formed after one month. Analysis calc. of C_29_H_53_NO_2_: C 77.79, H 11.93, N 3.13%, found: C 77.50, H 11.84, N 3.08%; FTIR (*ν*/cm^−1^): 2919, 2848, 2675, 2594, 2183, 1650, 1567, 1508, 1461, 1411, 1334, 1309, 1272, 1239, 1201, 1115, 1095, 1056, 1028, 932, 921, 750, 722, 650. Crystal data: *M* = 447.72 g mol^−1^, 0.36 × 0.08 × 0.05 mm^3^, monoclinic, space group *P*2_1_ (no. 4), *a* = 4.5934(6) Å, *b* = 56.597(9) Å, *c* = 5.5096(6) Å, *β* = 99.130(10)°, *V* = 1414.2(3) Å^3^, *Z* = 2, *D*_c_ = 1.051 g cm^−3^, *F*_000_ = 500.0, Mo Kα radiation, *λ* = 0.71073 Å, *T* = 120 K, 2*θ*_max_ = 53.97°, 14 179 reflections collected, 5663 unique (*R*_int_ = 0.1264). Final GooF = 1.026, *R*_1_ = 0.1156, w*R*_2_ = 0.2163, *R* indices based on 5663 reflections with *I* ≥ 2*σ*(*I*) (refinement on *F*^2^), 293 parameters, 22 restraints, *μ* = 0.064 mm^−1^.

## Conflicts of interest

There are no conflicts to declare.

## Supplementary Material

SC-011-D0SC02540B-s001

SC-011-D0SC02540B-s002
