# Supplementary material for: The crystal engineering of radiation-sensitive diacetylene cocrystals and salts
Source: Chem Sci. 2020 Jul 20;11(30):8025–35. doi: 10.1039/d0sc02540b (PMC8163068; doi:10.1039/d0sc02540b)
Supplement: SC-011-D0SC02540B-s001 [file SC-011-D0SC02540B-s001.pdf]

Electronic Supplementary Material (ESI) for Chemical Science.
This journal is © The Royal Society of Chemistry 2020

The Crystal Engineering of Radiation-Sensitive Diacetylene Cocrystals and Salts

Amy V. Hall[a], Dmitry S. Yufit[a], David C. Apperley[a], Larry Senak[b], Osama M. Musa[b], David K. Hood[b], and Jonathan W. Steed*[a]

Supplementary Information

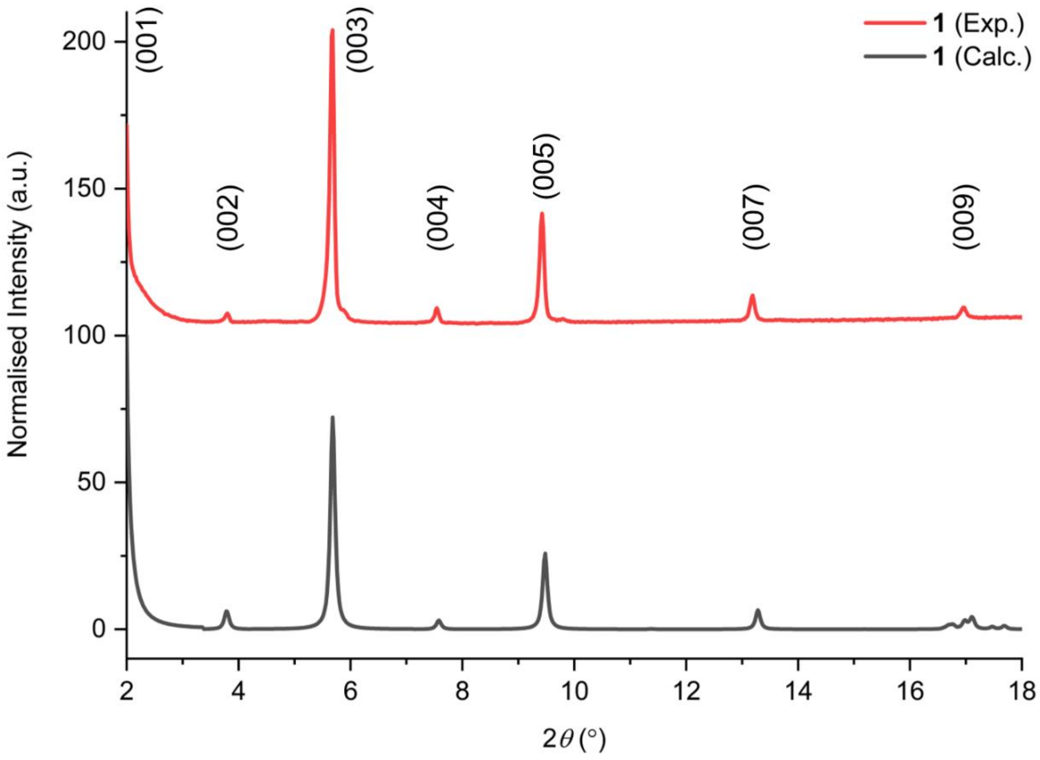

Figure S1. The experimental and calculated PXRD patterns of **1** highlighting the initial (00*l*) reflections. The experimental data were collected at room temperature (approx. 293 K), with the calculated data collected at 100 K.

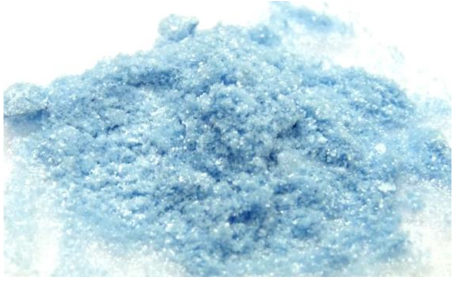

Figure S2. A photograph of **1** before any irradiation studies. Highlighting that the surface colouration of the crystals is not representative of the bulk material (no evidence of polymerisation).

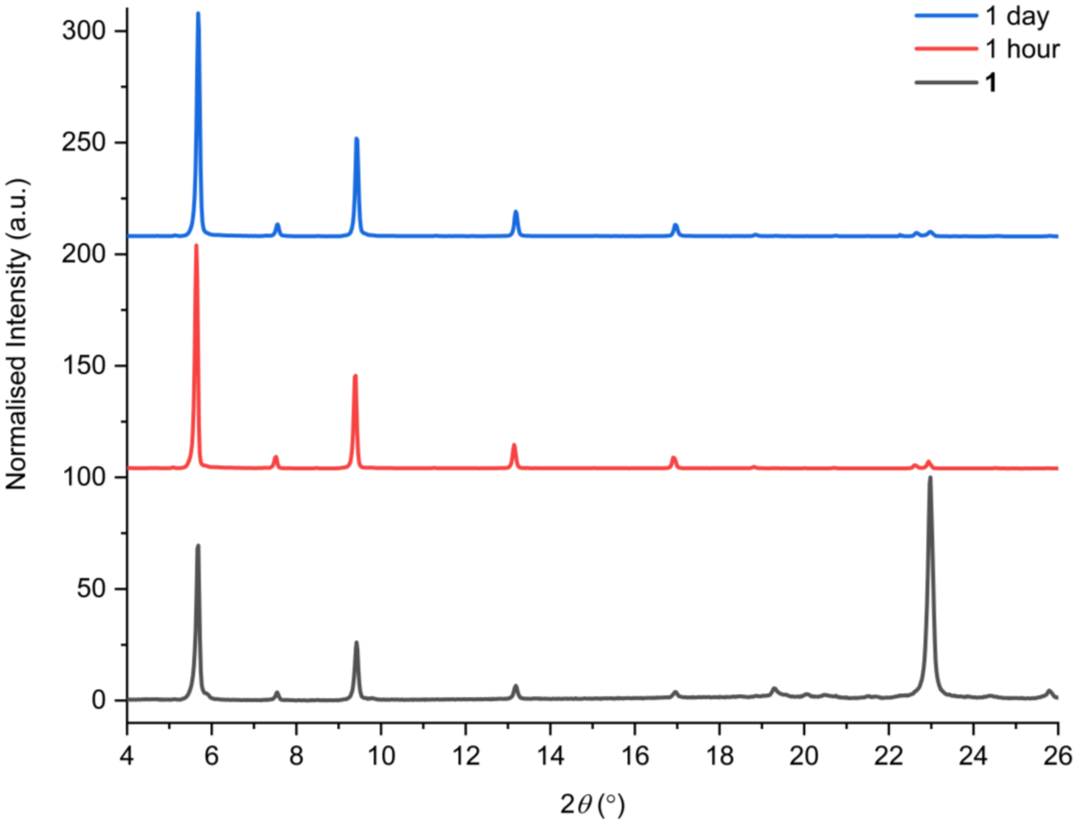

Figure S3. The experimental PXRD patterns of **1** irradiated for one hour and one day. The relative intensities are affected by differences in preferred orientation between the irradiated and non-irradiated samples.

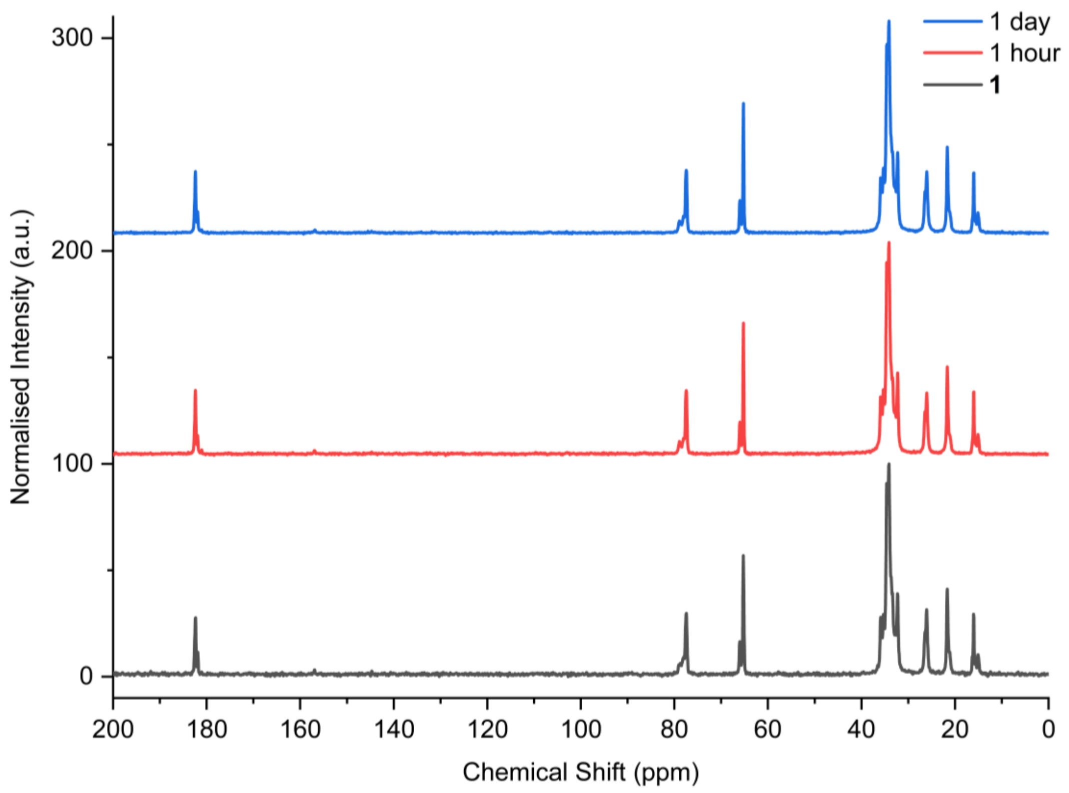

Figure S4. CP-MAS ^{13}C NMR spectra of **1** irradiated for different durations by UV light at 254 nm.

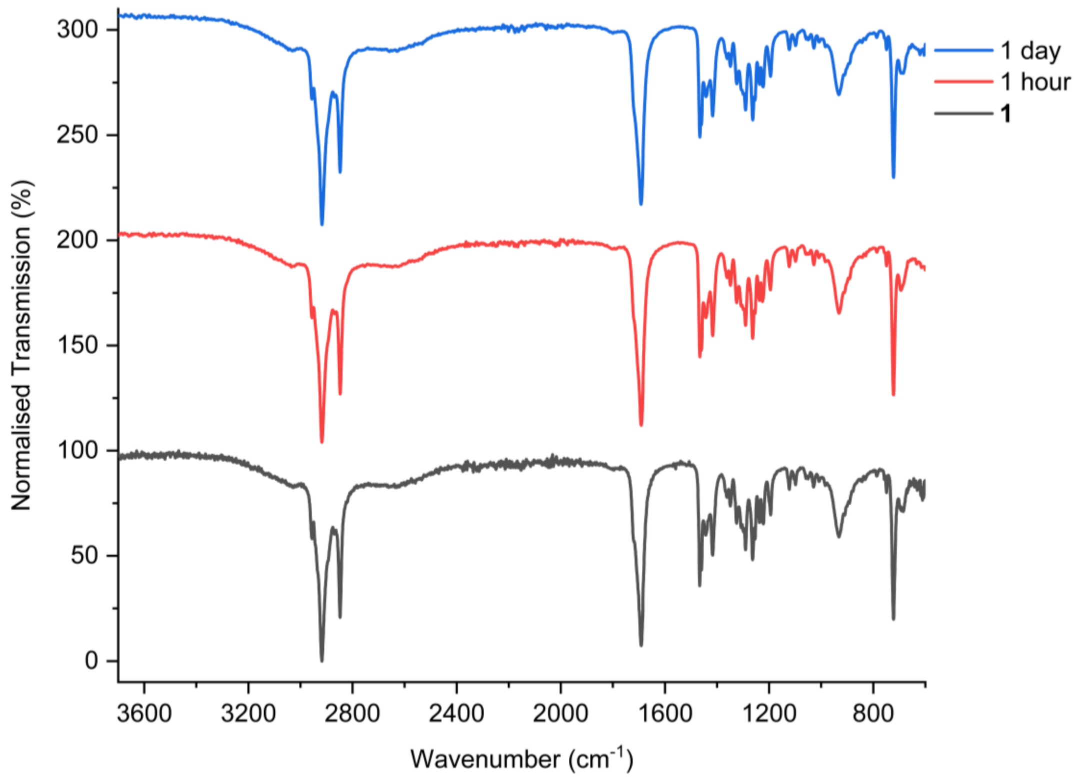

Figure S5. The FTIR spectra of **1** irradiated for different durations by UV light at 254 nm.

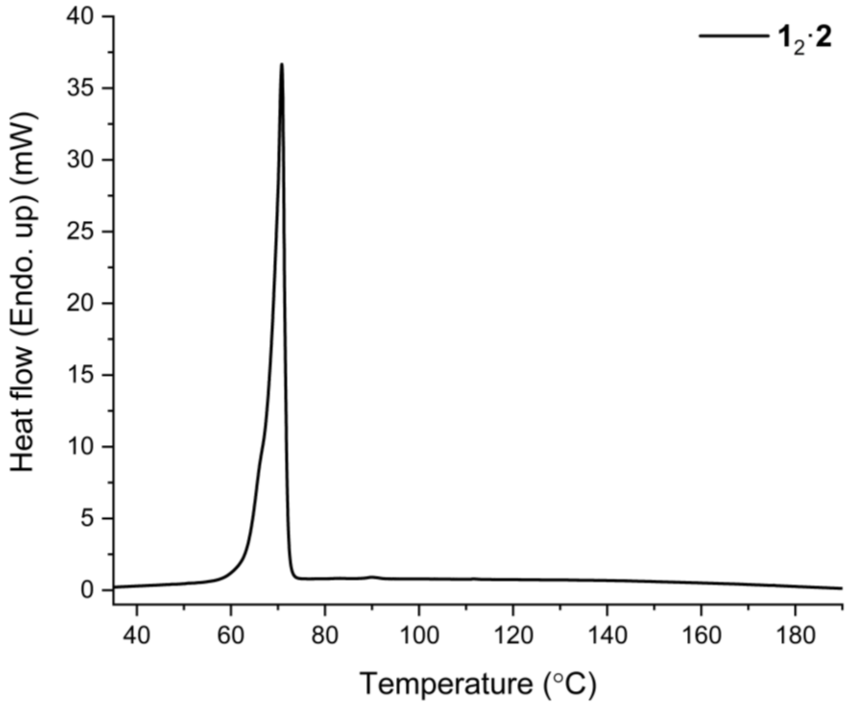

Figure S6. The DSC thermogram of $\mathbf{1}_2{\cdot}\mathbf{2}$ with a peak onset endotherm at 56.9 °C.

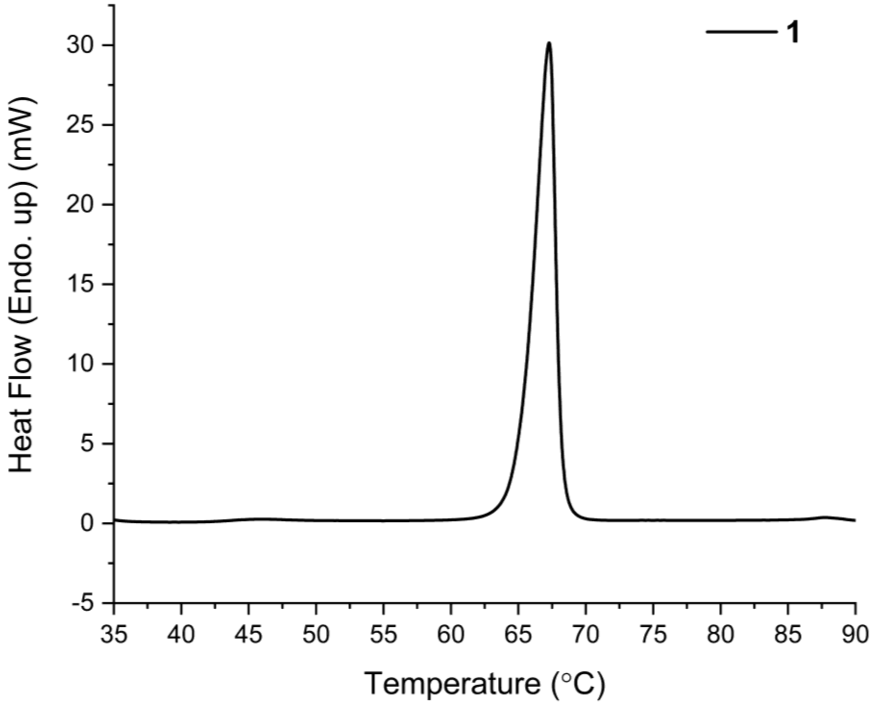

Figure S7. The DSC thermogram of **1** with a melt onset endotherm at 62.3 °C.

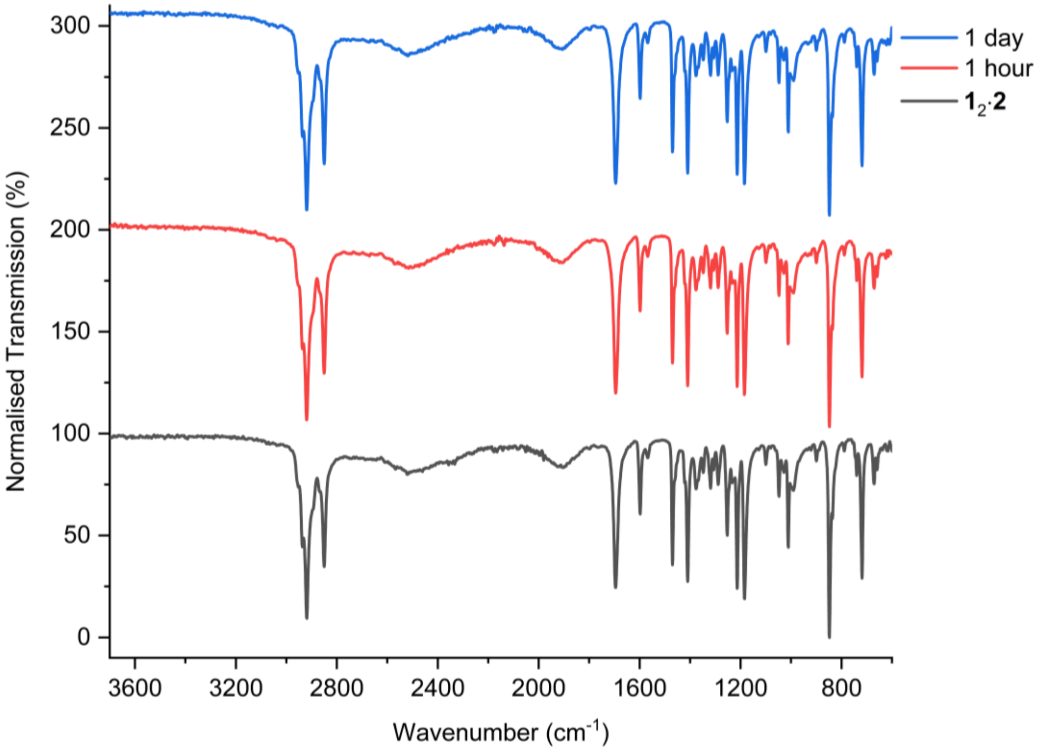

Figure S8. The FTIR spectra of $\mathbf{1}_2\cdot\mathbf{2}$ irradiated for one hour by UV light at 365 nm.

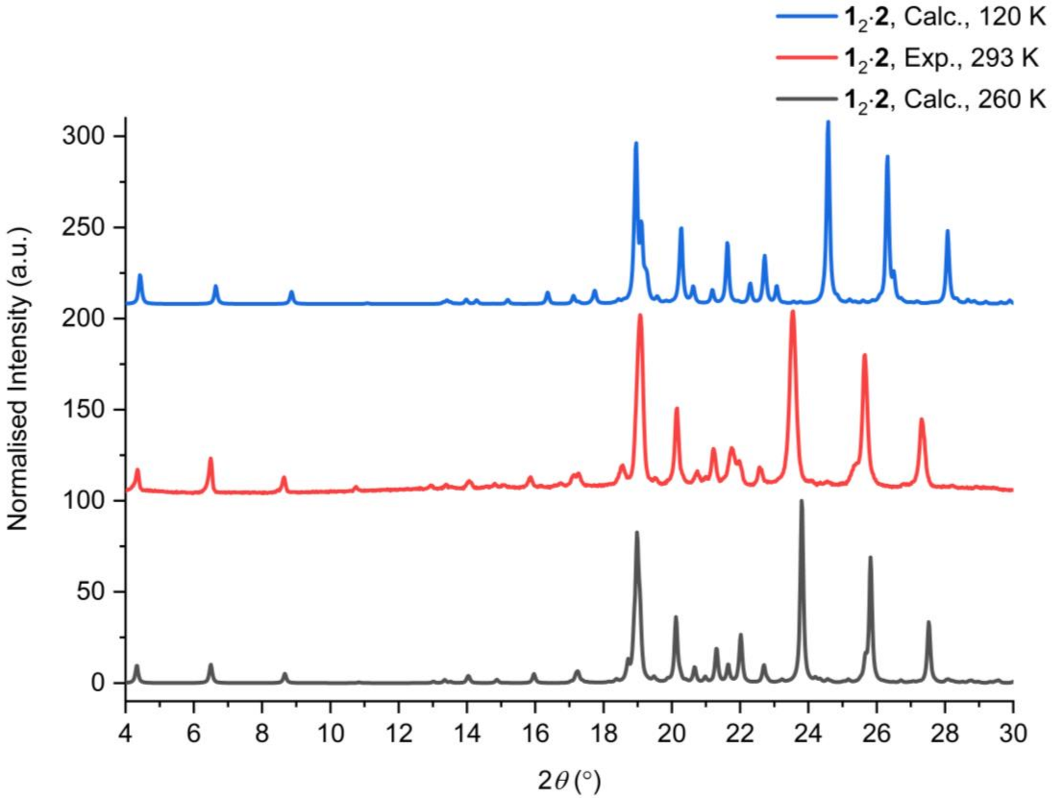

Figure S9. The calculated and experimental PXRD pattern of $\mathbf{1}_2\cdot\mathbf{2}$ at different temperatures.

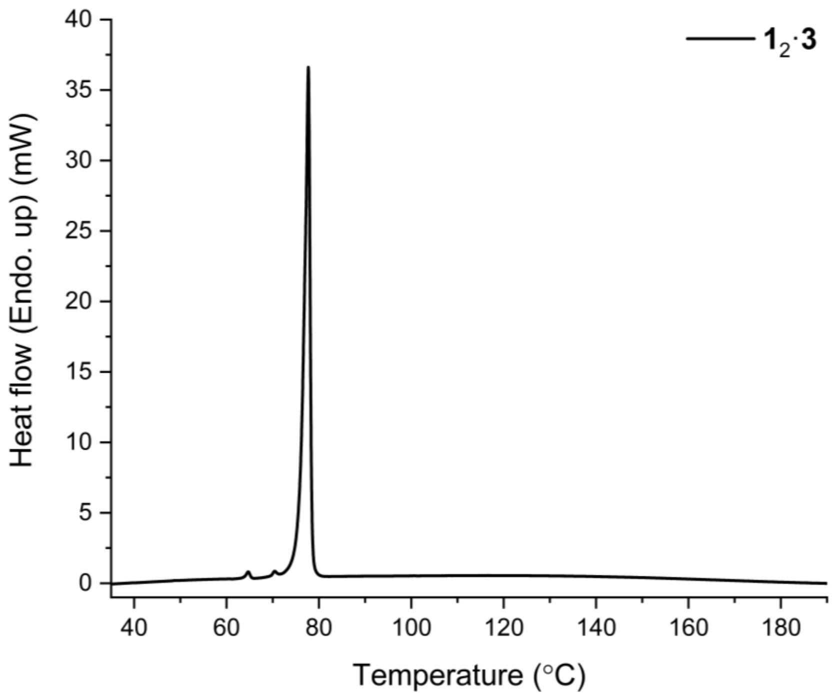

Figure S10. The DSC thermogram of **1**$_2$·**3** with a melt onset endotherm at 73.4 °C, with a residual peak of **1** at 63.7 °C.

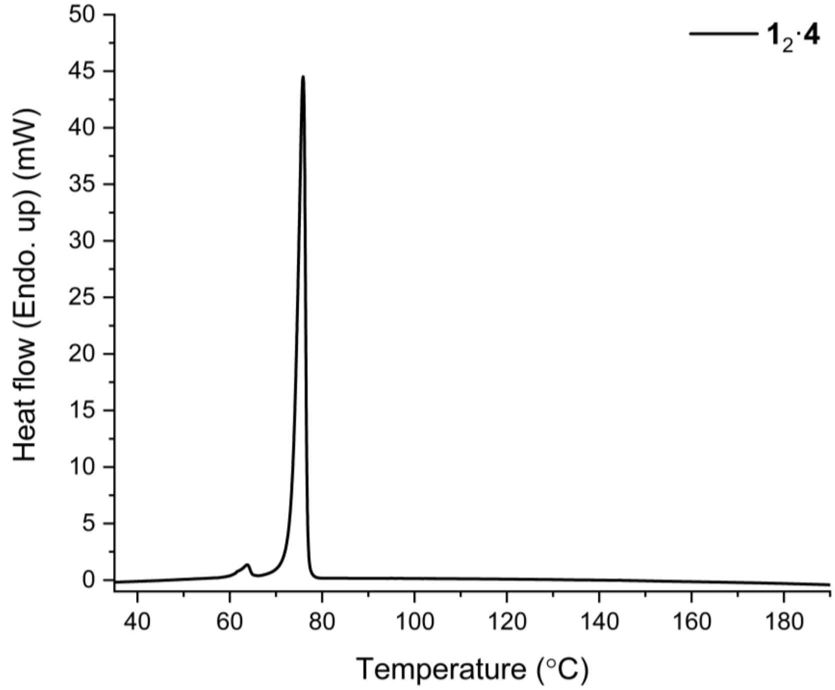

Figure S11. The DSC thermogram of **1**$_2$·**4** displaying a melt onset endotherm at 72.2 °C, with residual unreacted **1** at 62.6 °C.

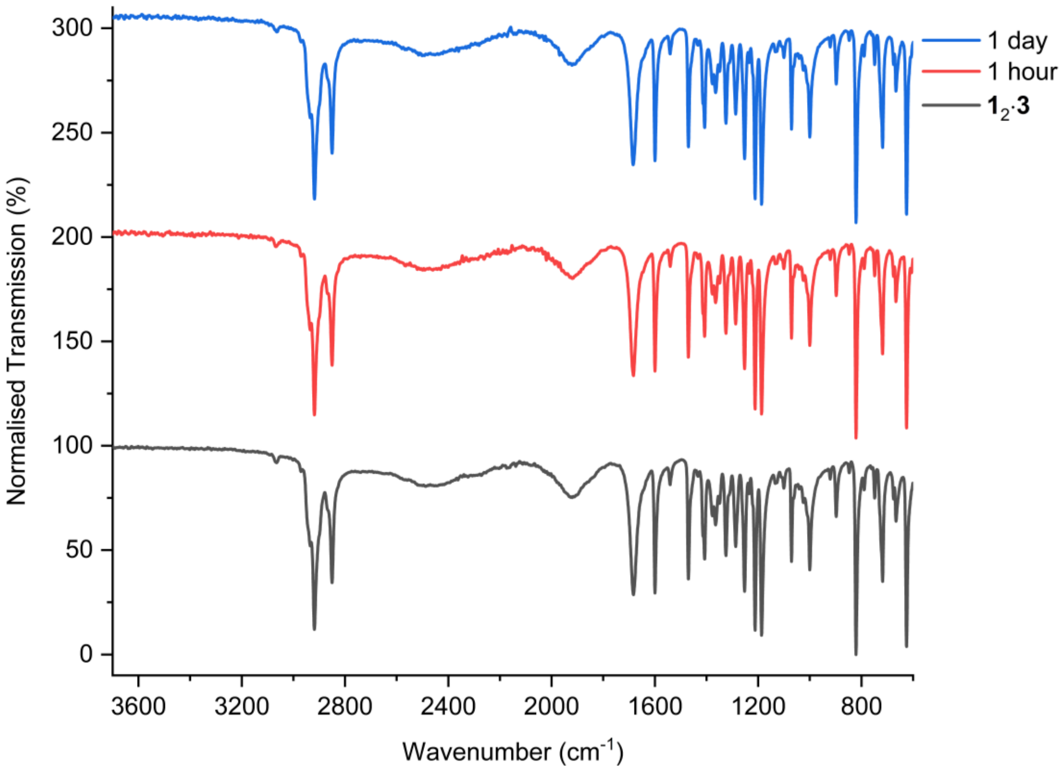

Figure S12. The FTIR spectra of $\mathbf{1}_2$·**3** irradiated for different durations by UV light at 254 nm.

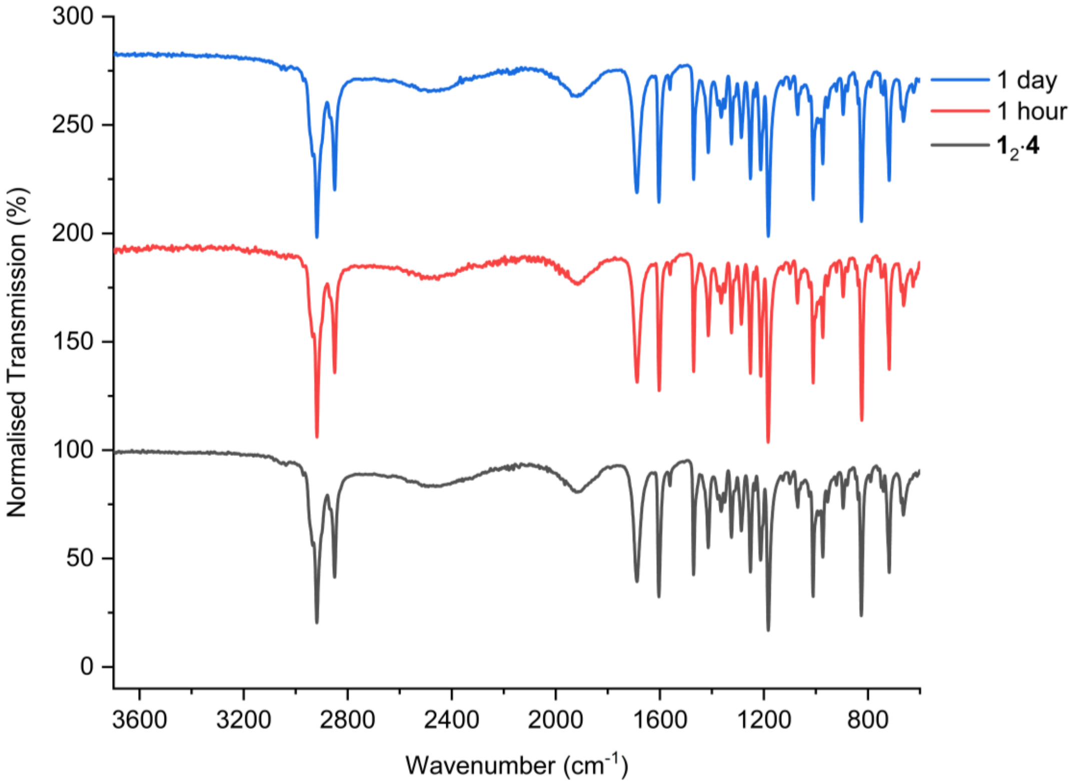

Figure S13. The FTIR spectra of $\mathbf{1}_2$·**4** irradiated for different durations by UV light at 254 nm.

Cocrystal	Temp.	a / Å	b / Å	c / Å	β / °	Volume / Å³
**1**$_2$·**3**	100 K	5.4415(2)	8.9535(4)	55.673(3)	90.8823(10)	2712.1(2)
	260 K	5.4783(3)	8.9976(5)	56.951(4)	92.506(2)	2804.5(3)
	273 K	5.4761(3)	8.9950(5)	57.173(4)	92.710(2)	2813.0(5)
**1**$_2$·**4**	120 K	5.4494(3)	8.9235(5)	57.441(3)	92.643(2)	2790.2(3)
	260 K	5.4847(4)	8.9801(6)	58.562(4)	90.849(3)	2884.1(3)
	273 K	5.503(5)	9.023(7)	58.93(5)	90.24(3)	2927.0(7)

Table S1. The unit cell axes of **1**$_2$·**3** and **1**$_2$·**4** collected at various temperatures.

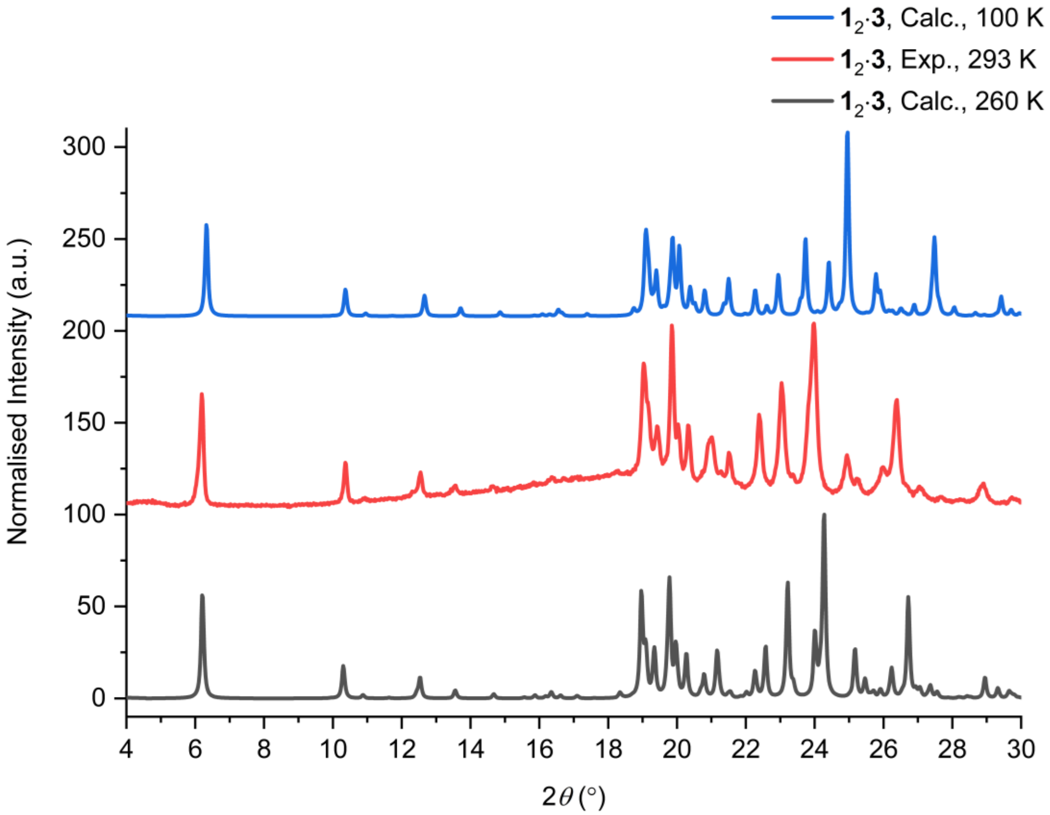

Figure S14. The calculated and experimental PXRD patterns of **1**$_2$·**3** at different temperatures.

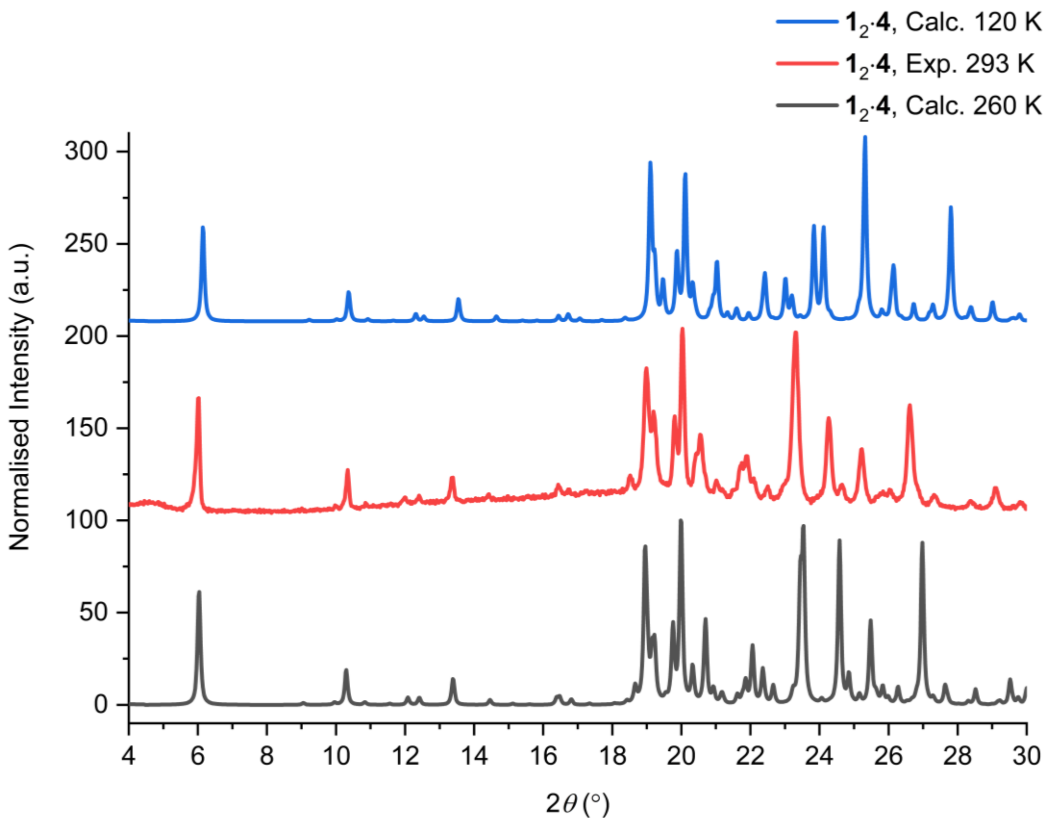

Figure S15. The calculated and experimental PXRD patterns of **1**$_2$·**4** collected at different temperatures.

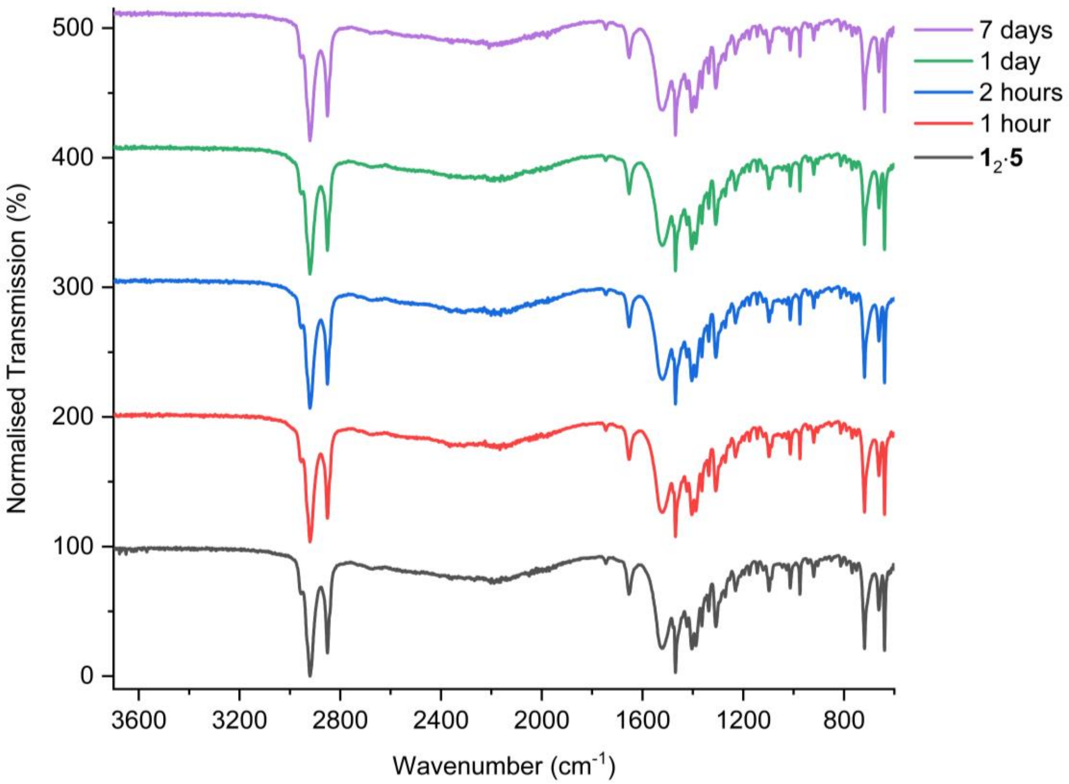

Figure S16. The FTIR spectra of **1**$_2$·**5** irradiated for different durations by UV light at 254 nm.

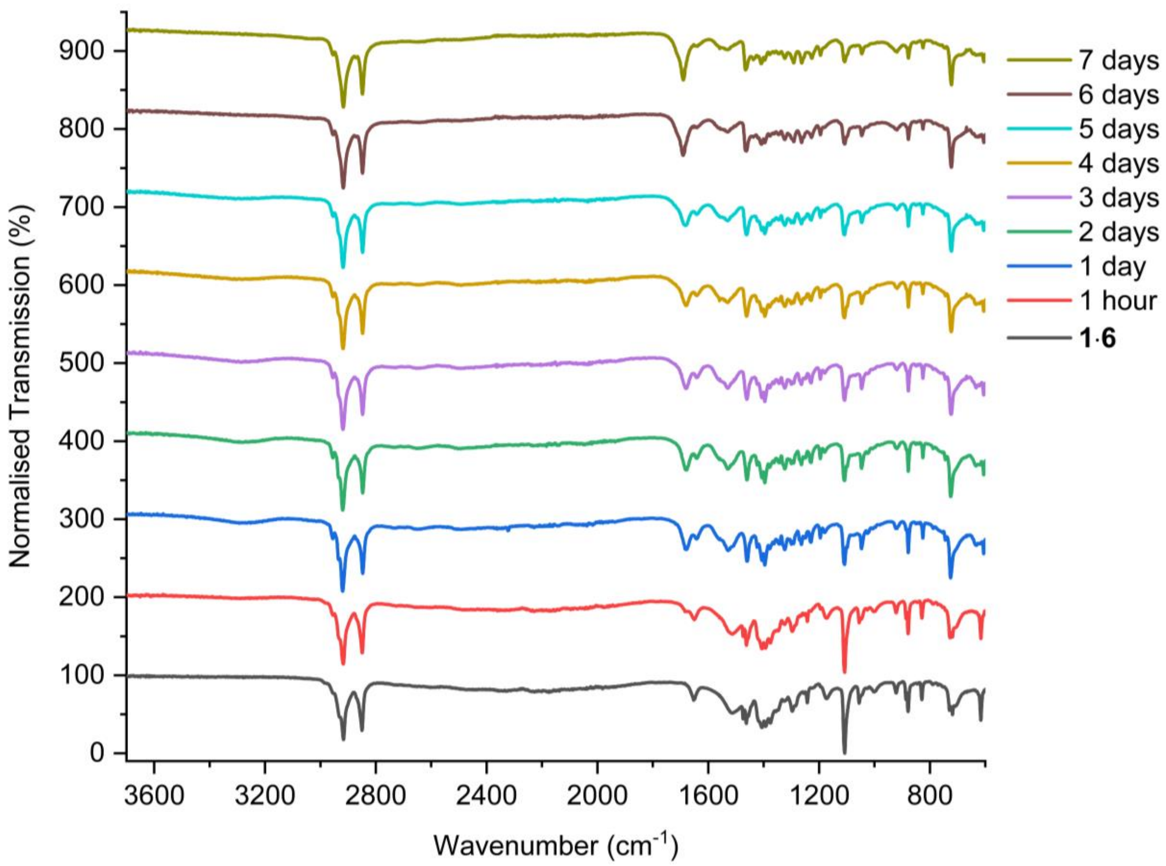

Figure S17. The FTIR spectra of **1·6** irradiated for different durations by UV light at 254 nm.

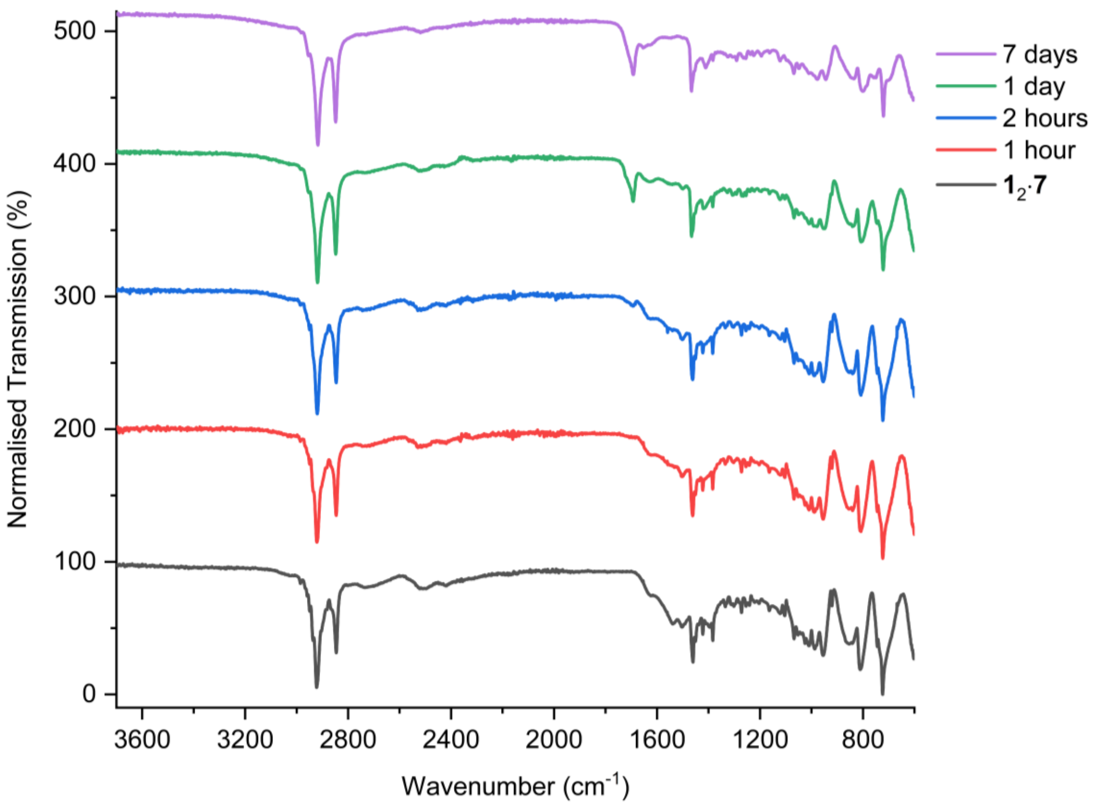

Figure S18. The FTIR spectra of $\mathbf{1}_2\cdot\mathbf{7}$ irradiated for different durations by UV light at 254 nm.

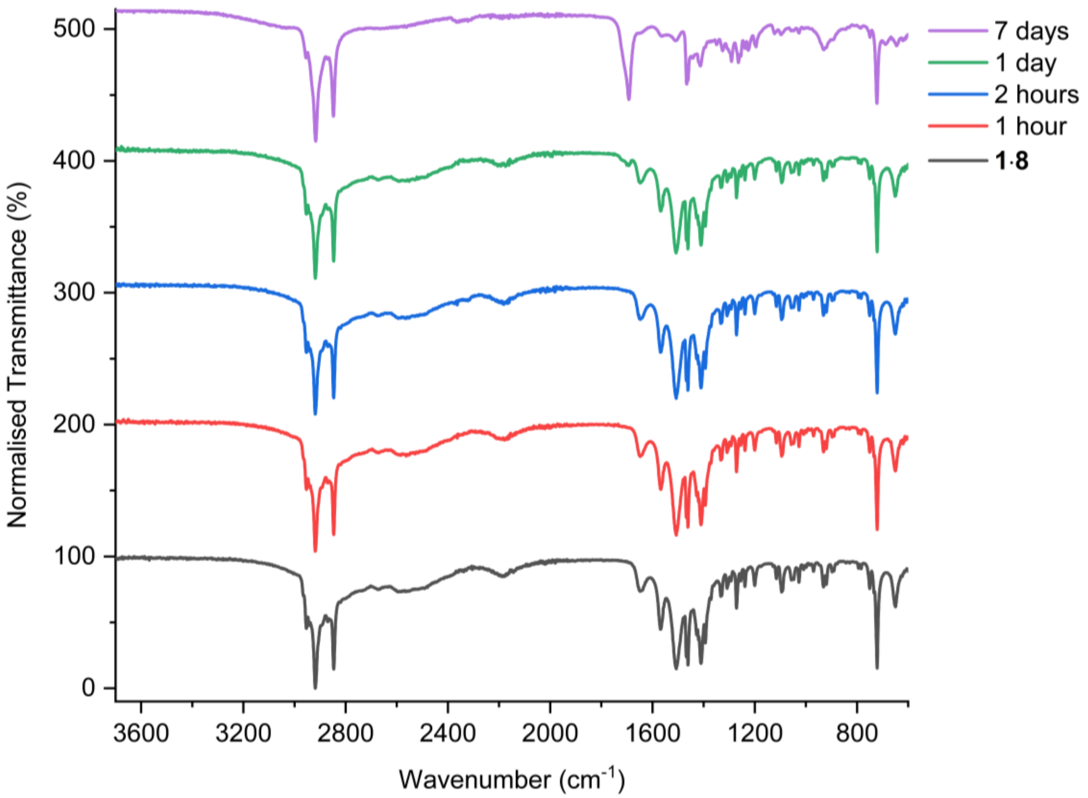

Figure S19. The FTIR spectra of **1·8** irradiated for different durations by UV light at 254 nm.

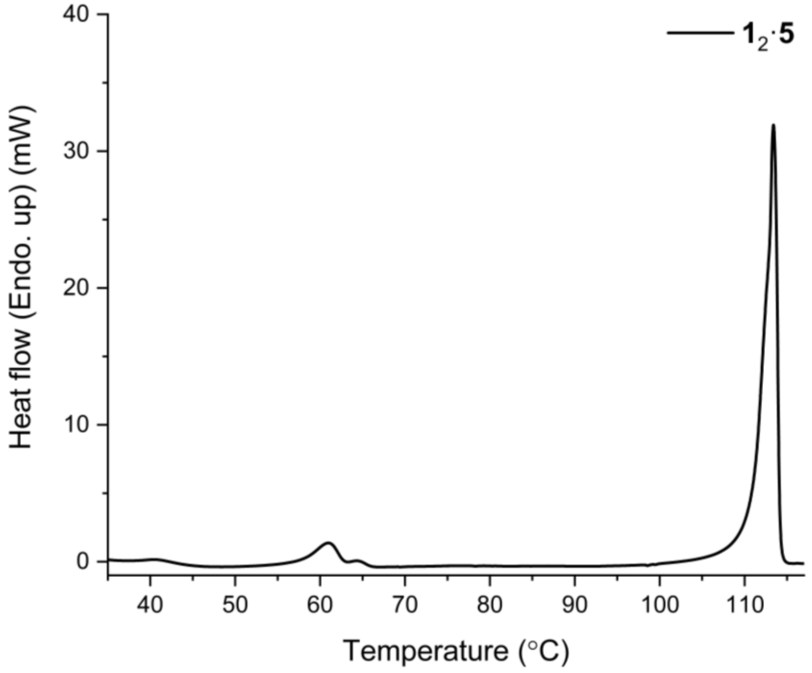

Figure S20. The DSC thermogram of $\mathbf{1_2{\cdot}5}$ displaying a melt onset endotherm at 107.4 °C with residual unreacted **1** between 59.5-64.6 °C.

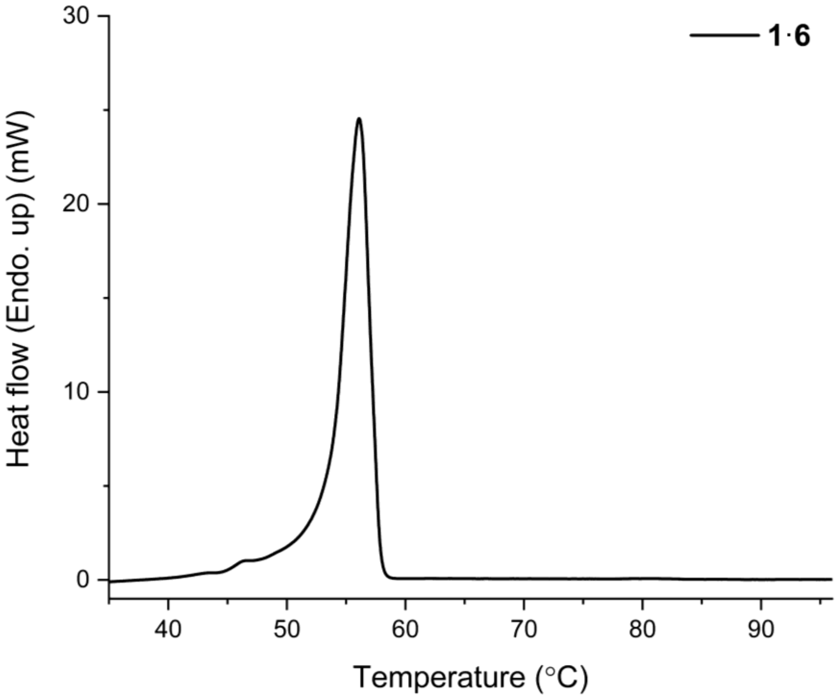

Figure S21. The DSC thermogram of **1·6** displaying a melt onset endotherm at 43.8 °C.

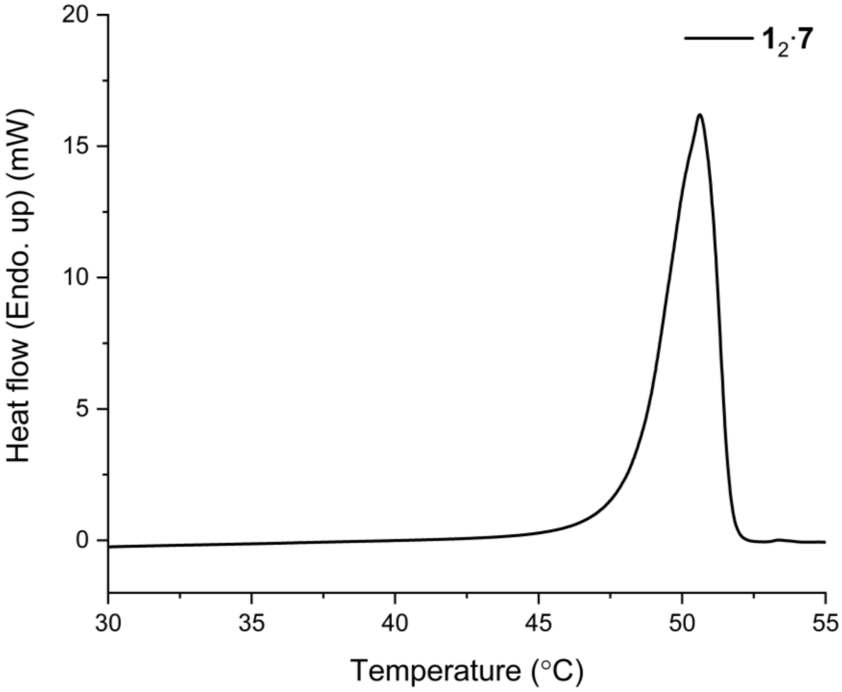

Figure S22. The DSC thermogram of **1**$_2$·**7** displaying a melt onset endotherm at 45.8 °C.

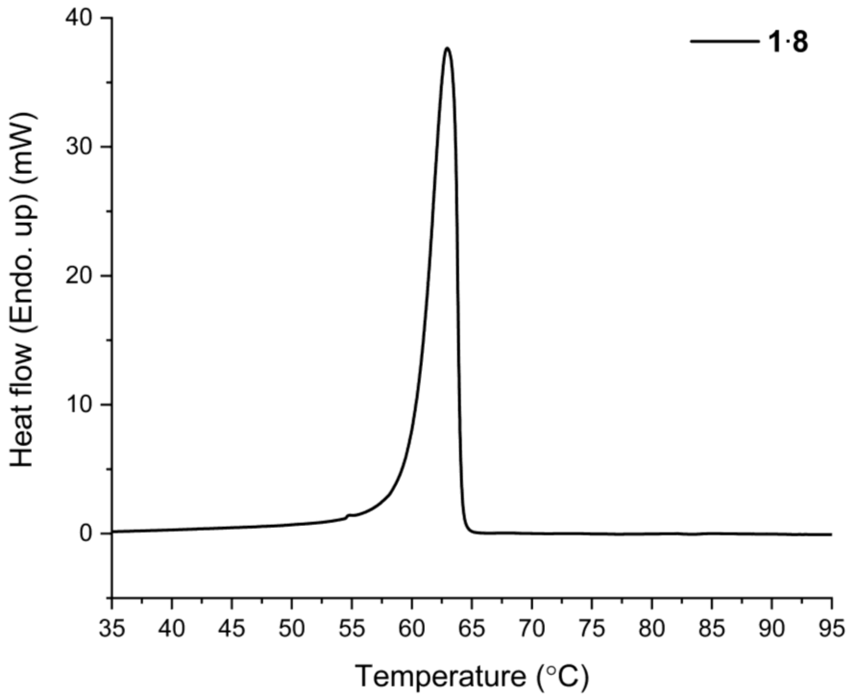

Figure S23. The DSC thermogram of **1·8** displaying a melt onset endotherm at 57.3 °C.

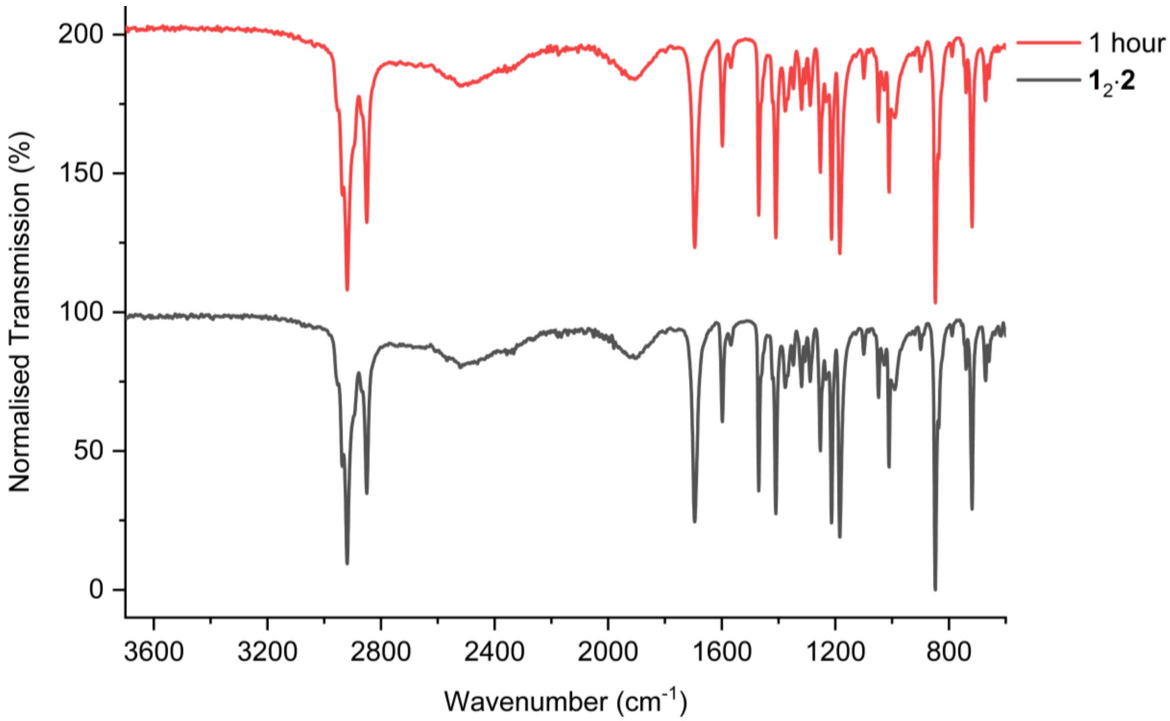

Figure S24. The FTIR spectra of $\mathbf{1}_2$·**2** irradiated for one hour by UV light at 254 nm.

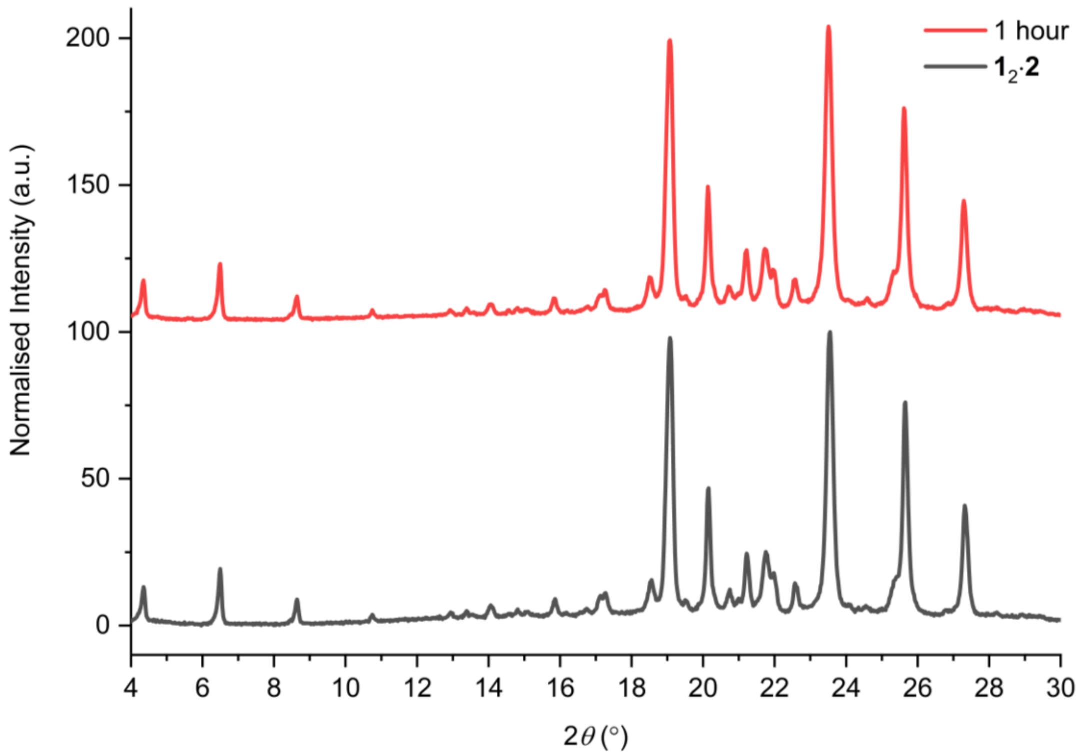

Figure S25. The experimental PXRD patterns of $\mathbf{1}_2\cdot\mathbf{2}$ irradiated for one hour by UV light at 254 nm.

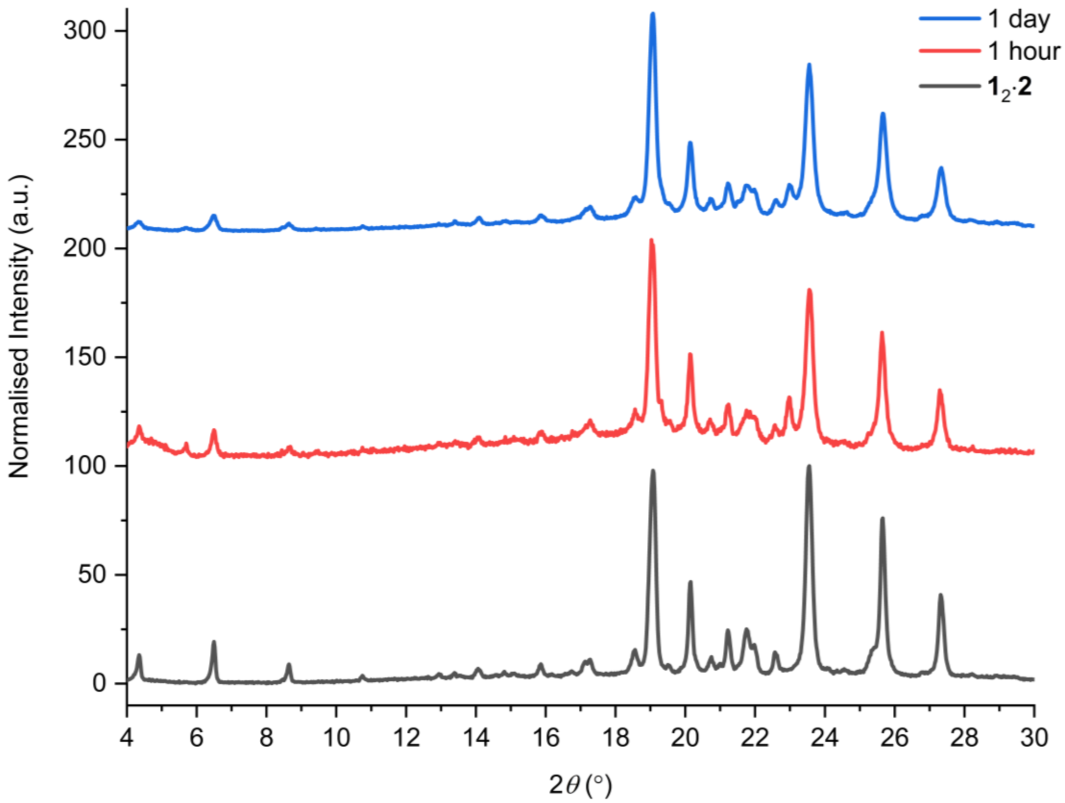

Figure S26. The experimental PXRD patterns of $\mathbf{1}_2\cdot\mathbf{2}$ irradiated for different durations by UV light at 365 nm. Additional peak at 23.0 2ϑ in the one hour and one day patterns correlate to **1**.

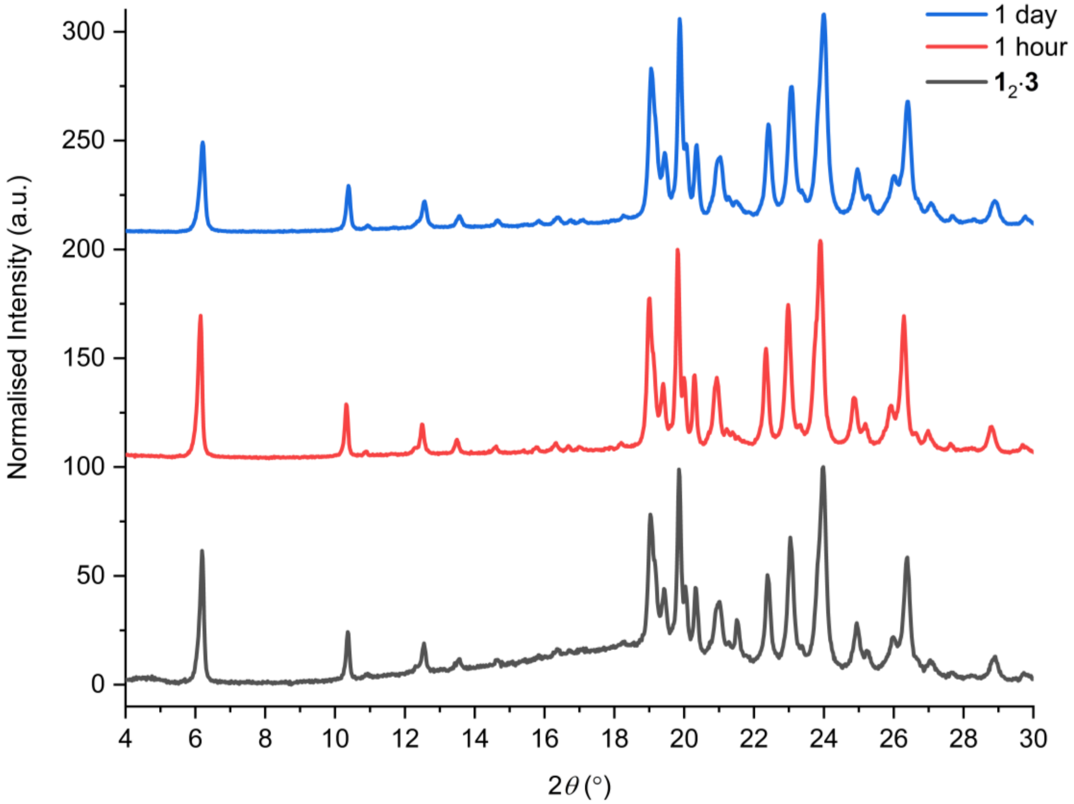

Figure S27. The experimental PXRD patterns of **1**$_2$·**3** irradiated for different durations by UV light at 254 nm.

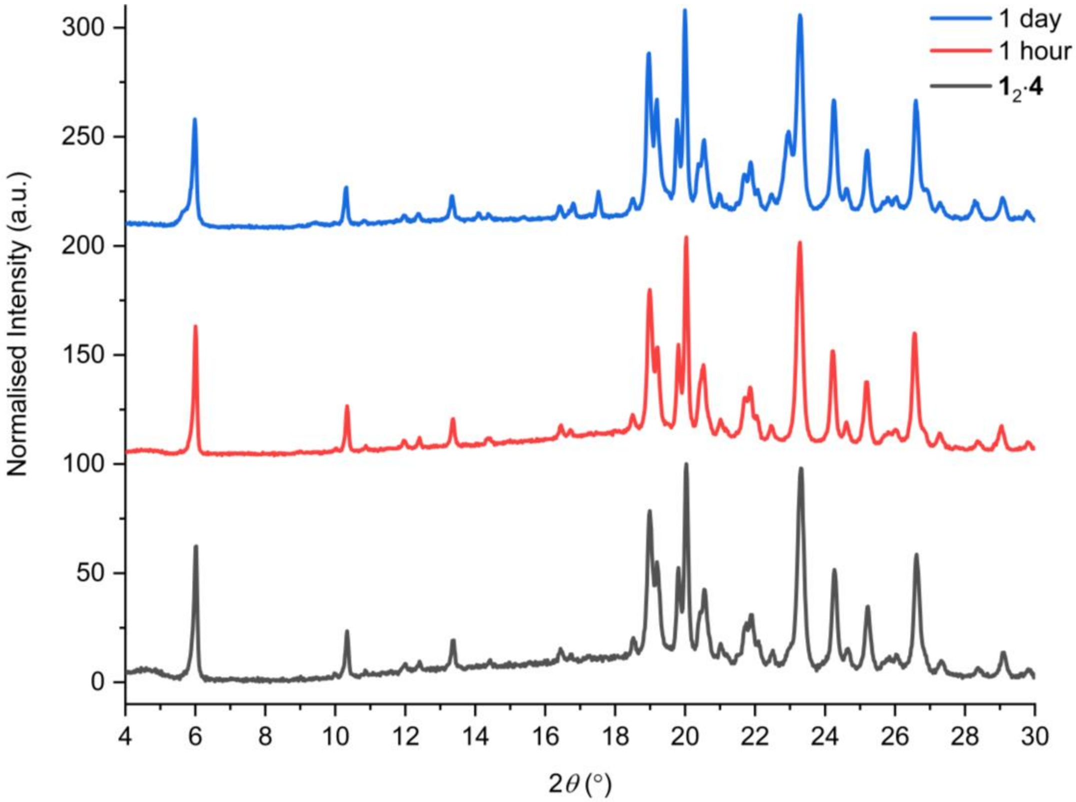

Figure S28. The experimental PXRD patterns of $\mathbf{1}_2{\cdot}\mathbf{4}$ irradiated for different durations by UV light at 254 nm. The peaks emerging in the one-day irradiated patterns at 5.7, 17.5, and 23.0 2ϑ, correlate to peaks of **1**.

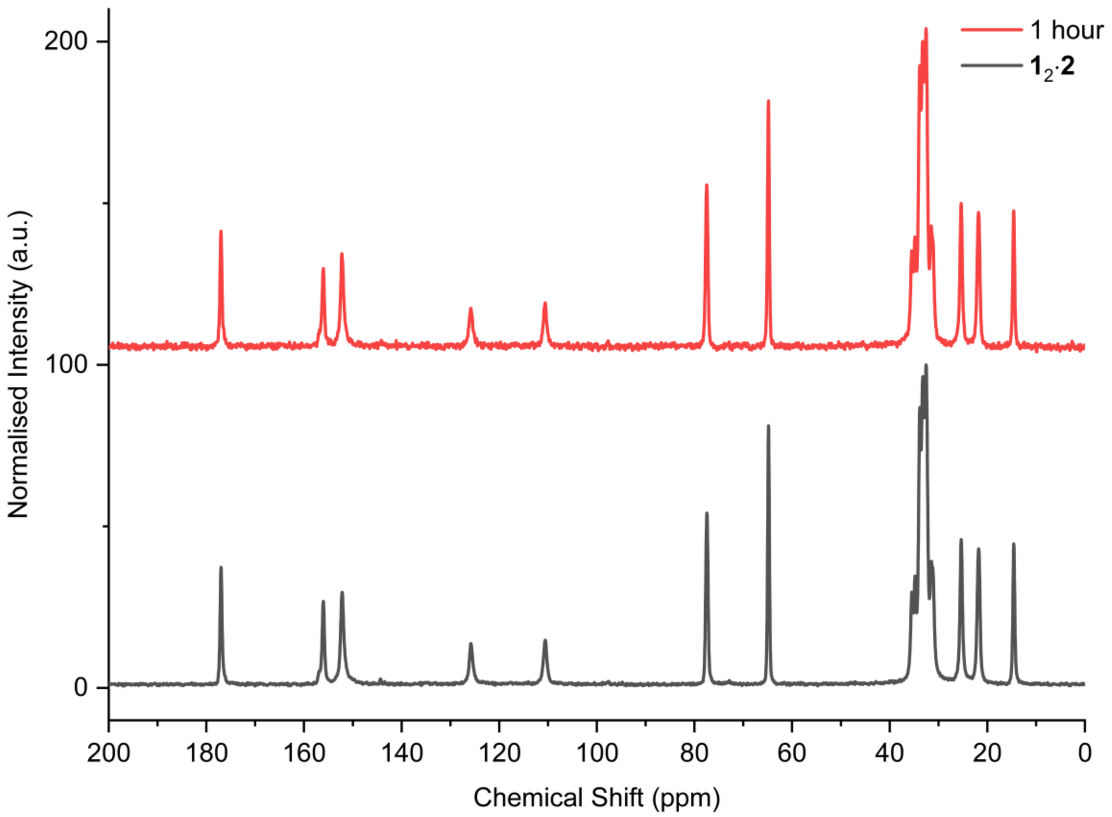

Figure S29. CP-MAS ^{13}C NMR spectra of $\mathbf{1}_2{\cdot}\mathbf{2}$ irradiated for different durations by UV light at 254 nm.

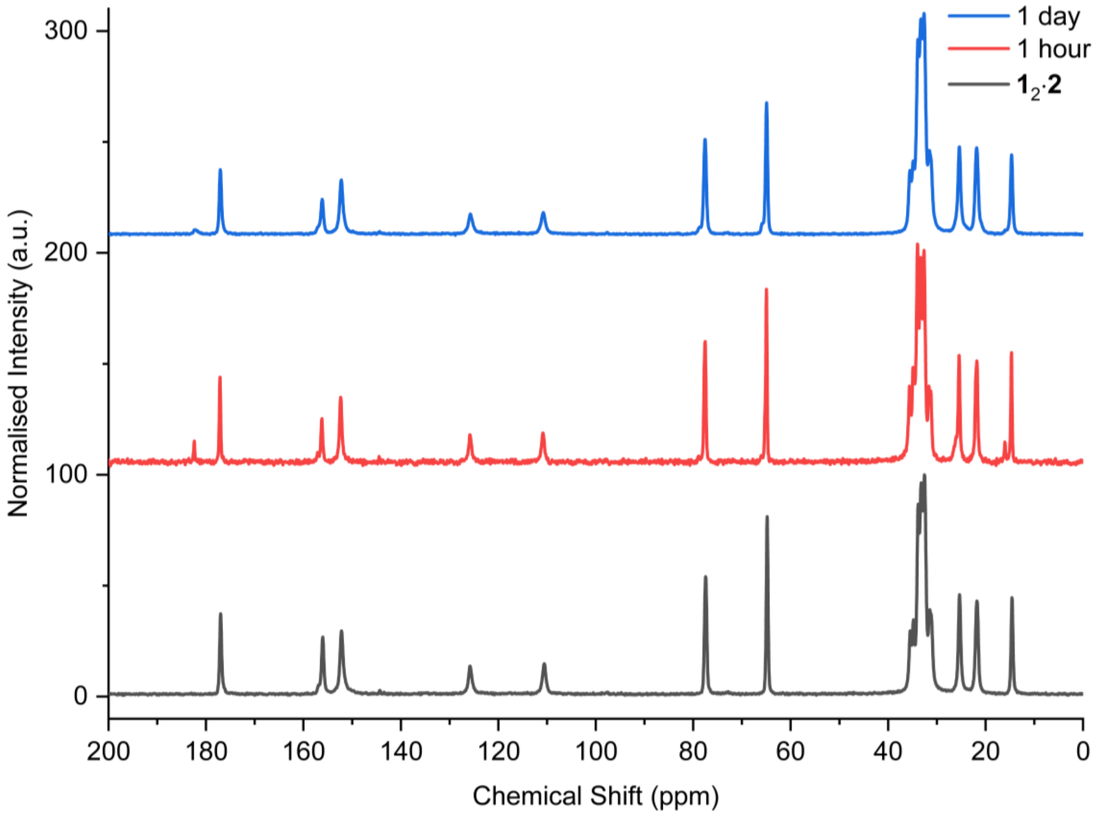

Figure S30. CP-MAS ^{13}C NMR spectra of $\mathbf{1}_2\cdot\mathbf{2}$ irradiated for one hour by UV light at 365 nm. The additional peak at 182.5 ppm which correlates to the carboxylate peak of **1**.

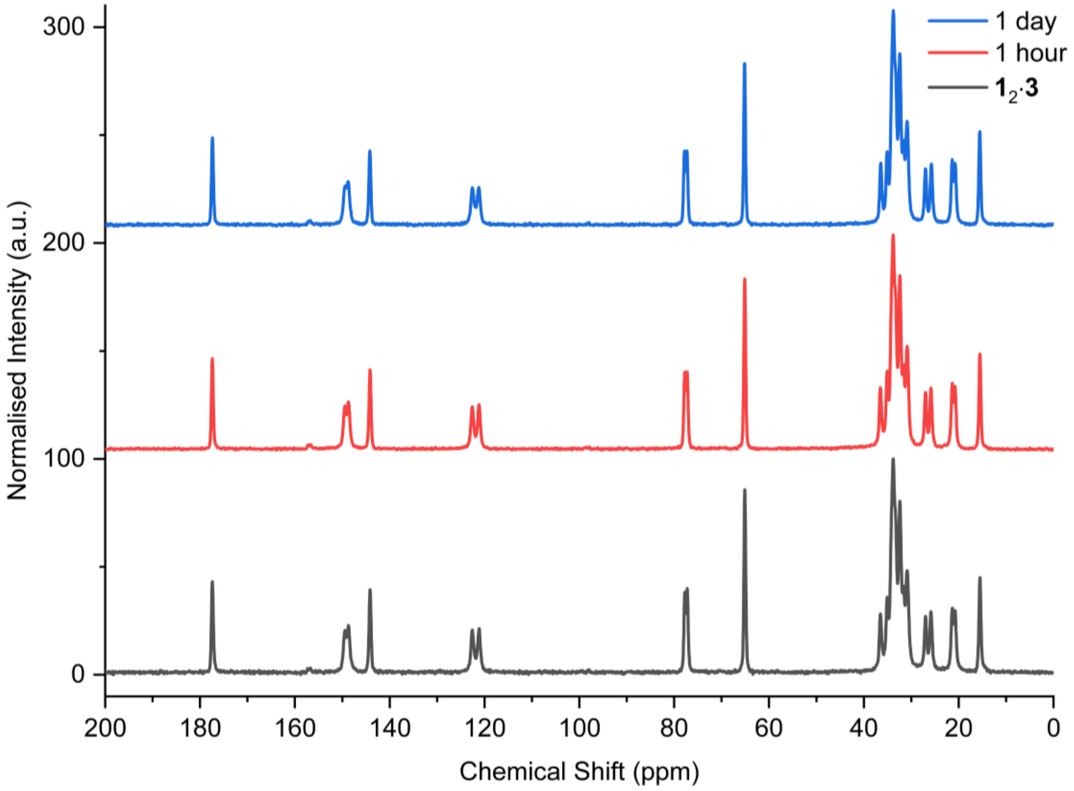

Figure S31. CP-MAS ^{13}C NMR spectra of $\mathbf{1}_2{\cdot}\mathbf{3}$ irradiated for different durations by UV light at 254 nm.

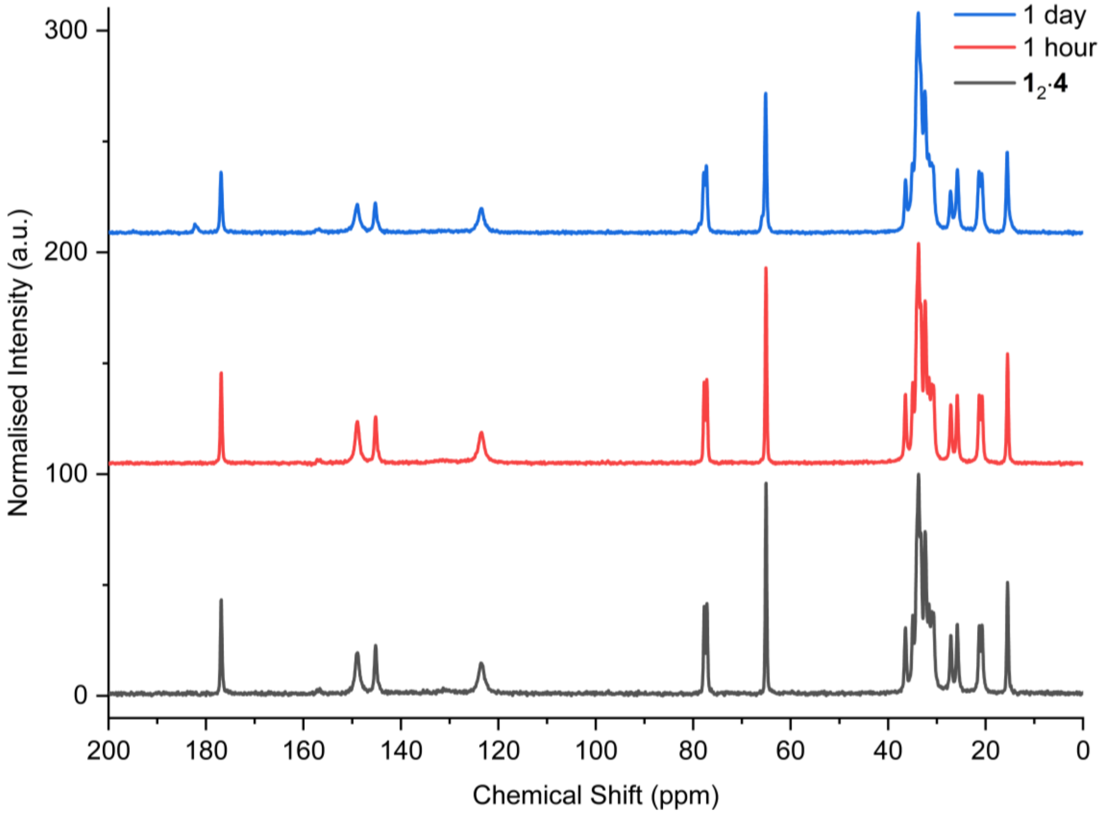

Figure S32. CP-MAS ^{13}C NMR spectra of **1**$_2$·**4** irradiated for different durations by UV light at 254 nm. The additional peak at 180.3 ppm in the one-day irradiated spectra corresponds to the carboxylate peak of **1**.

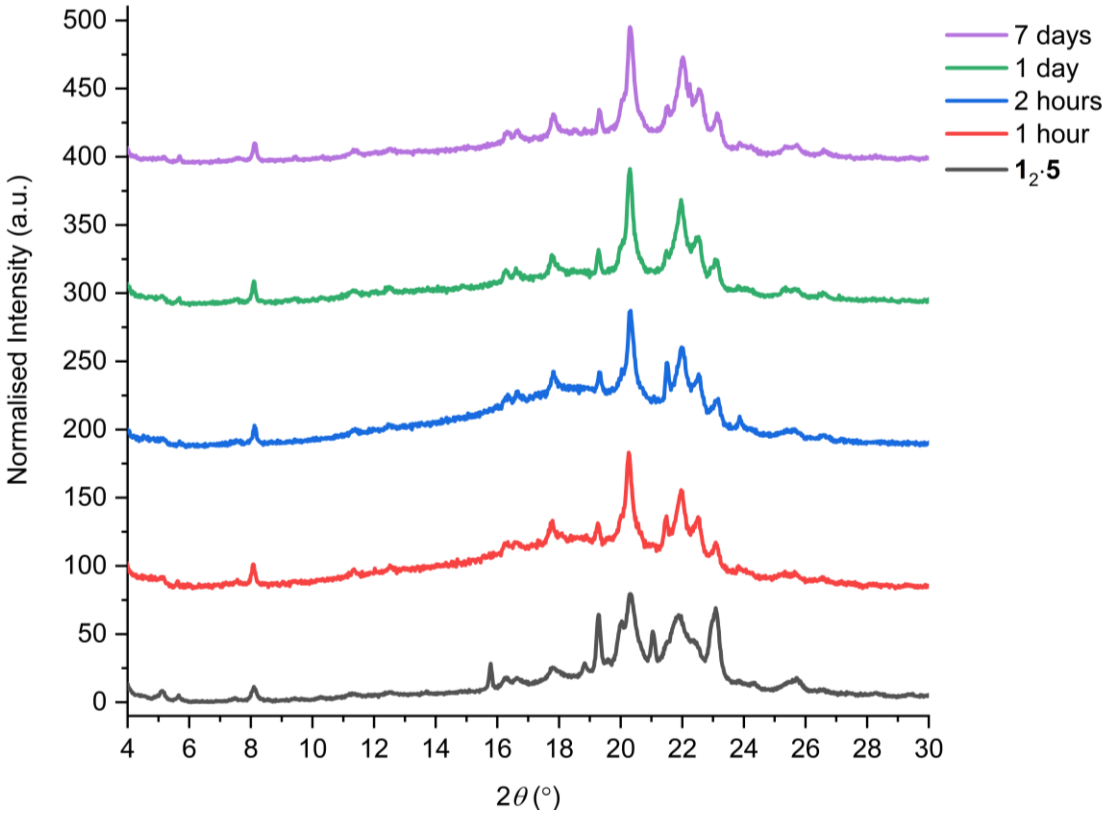

Figure S33. The experimental PXRD patterns of $\mathbf{1}_2{\cdot}\mathbf{5}$ irradiated for different durations by UV light at 254 nm.

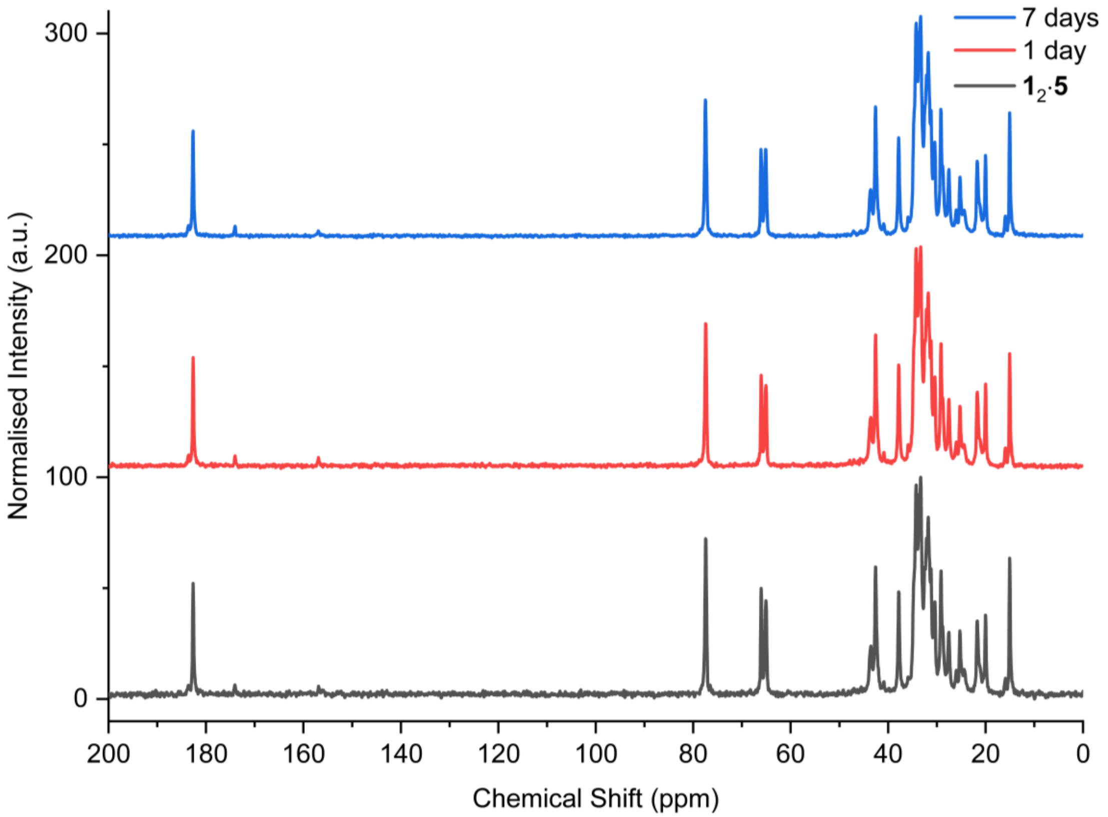

Figure S34. CP-MAS ^{13}C NMR spectra of **1**$_2$·**5** irradiated for different durations by UV light at 254 nm.

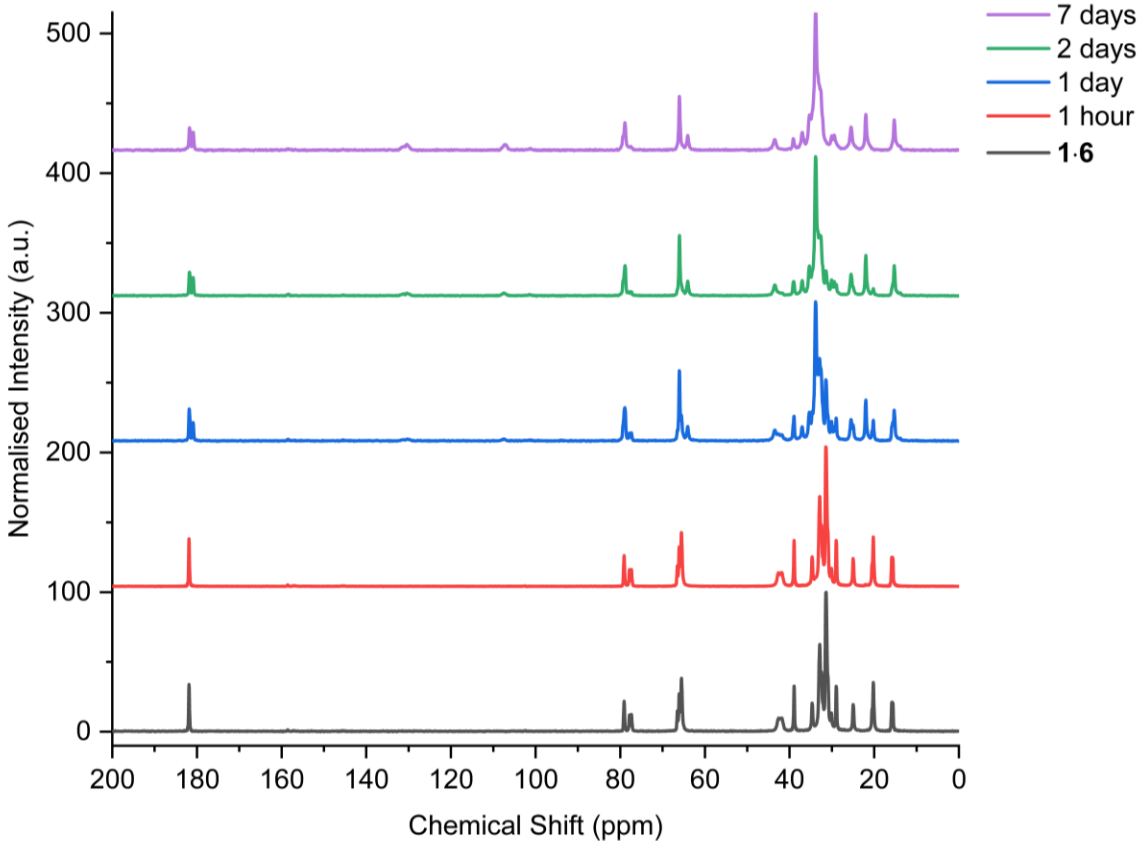

Figure S35. CP-MAS ^{13}C NMR spectra of **1·6** irradiated for different durations by UV light at 254 nm.

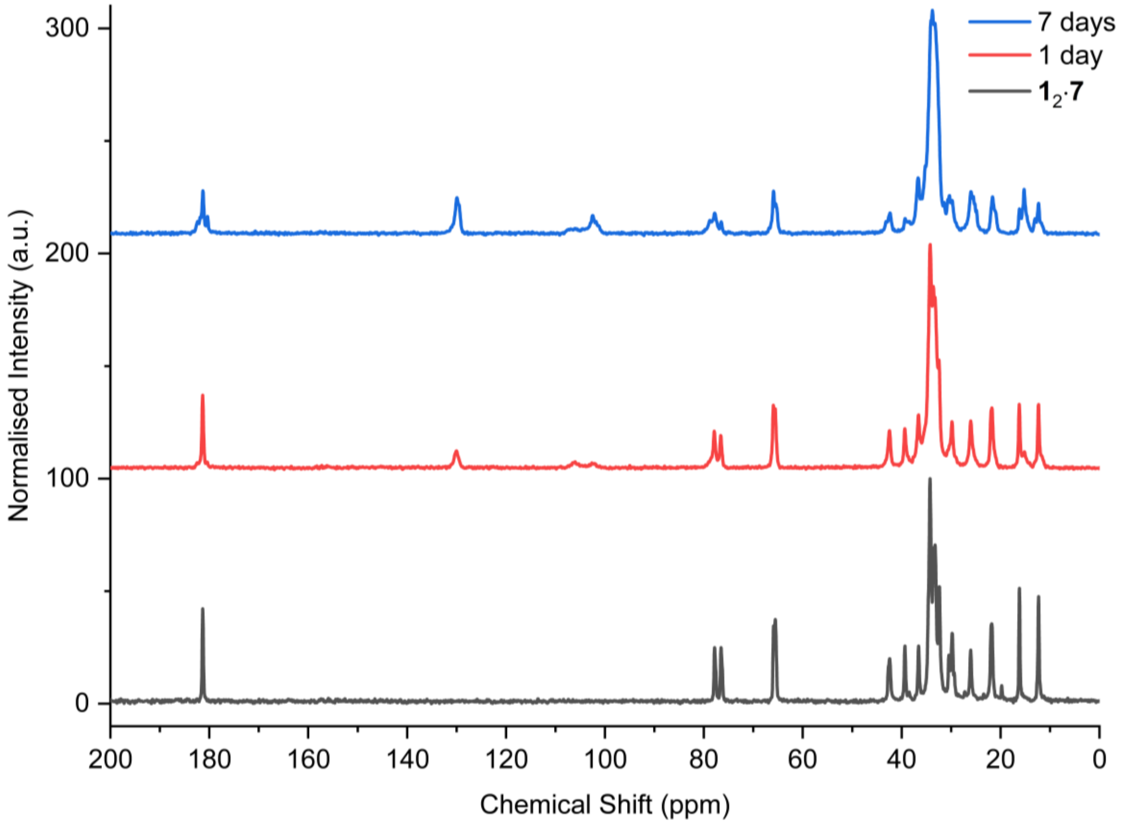

Figure S36. CP-MAS ^{13}C NMR spectra of $\mathbf{1}_2{\cdot}\mathbf{7}$ irradiated for different durations by UV light at 254 nm.

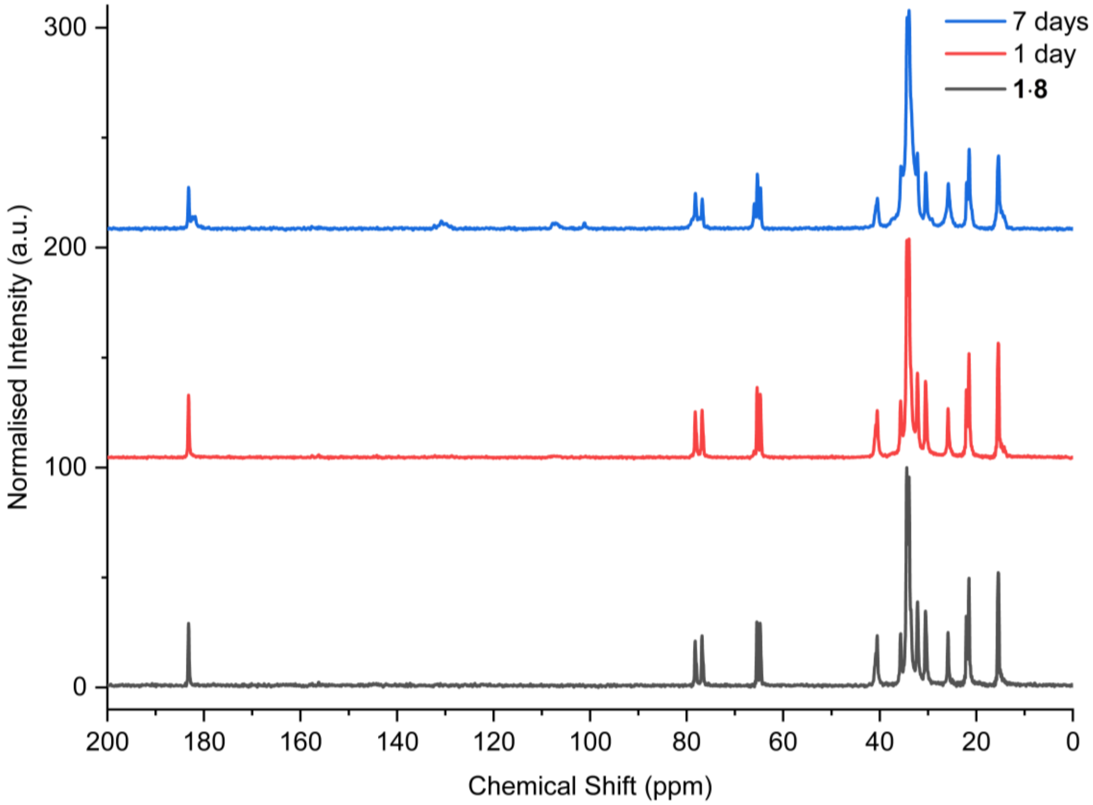

Figure S37. CP-MAS ^{13}C NMR spectra of **1·8** irradiated for different durations by UV light at 254 nm.

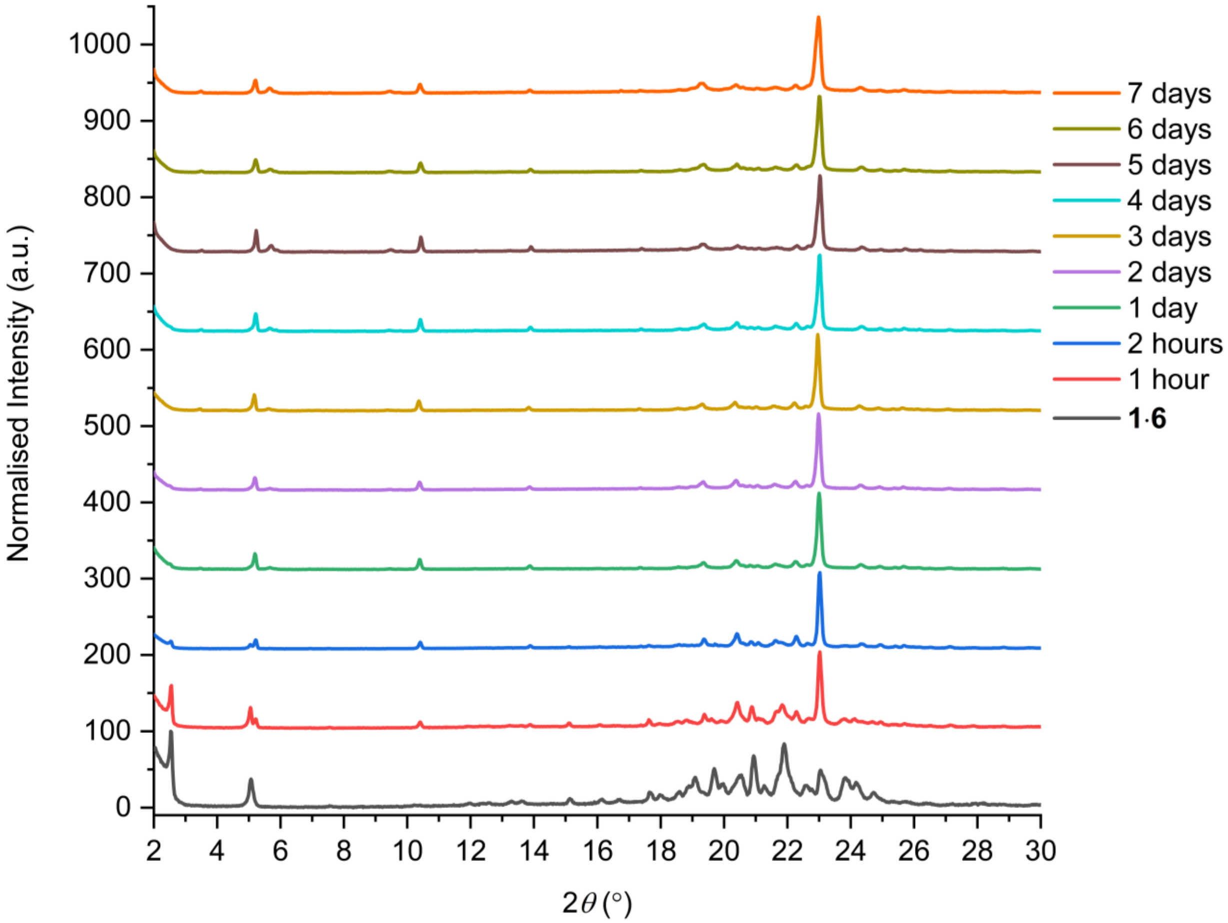

Figure S38. The experimental PXRD patterns of **1·6** irradiated for different durations by UV light at 254 nm.

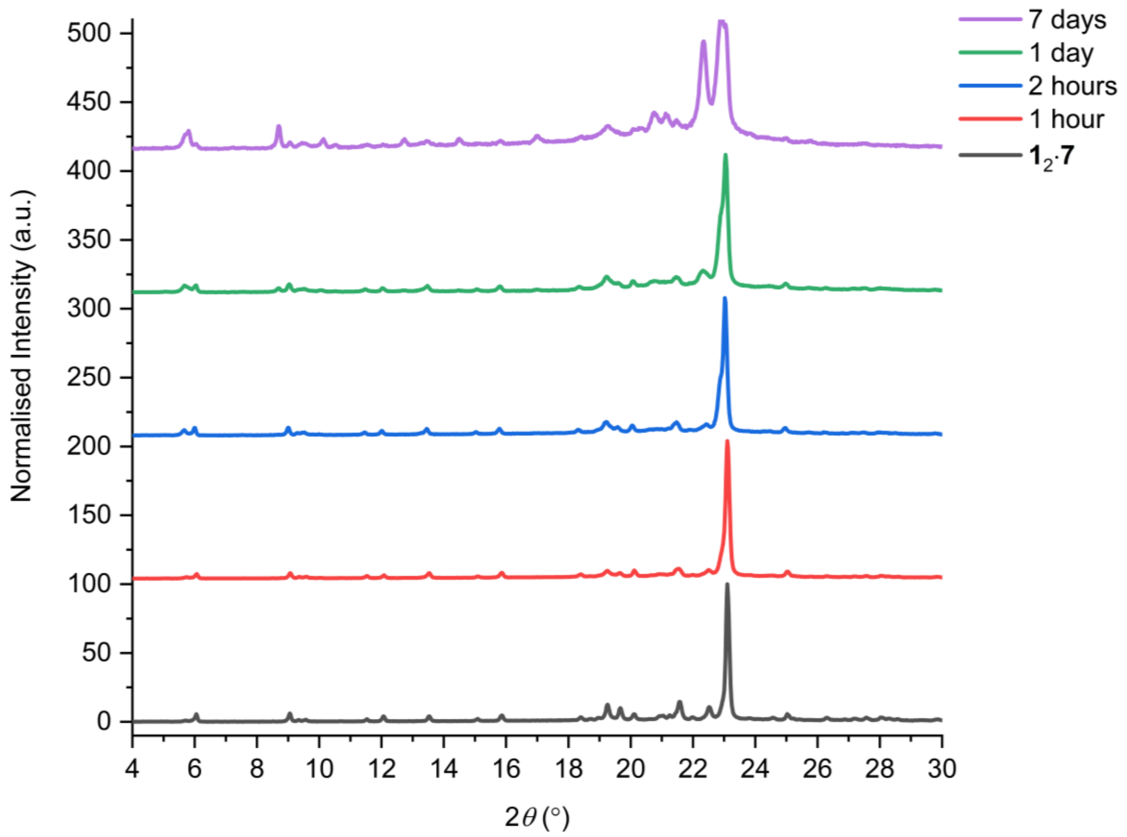

Figure S39. The experimental PXRD patterns of $\mathbf{1}_2{\cdot}\mathbf{7}$ irradiated for different durations by UV light at 254 nm.

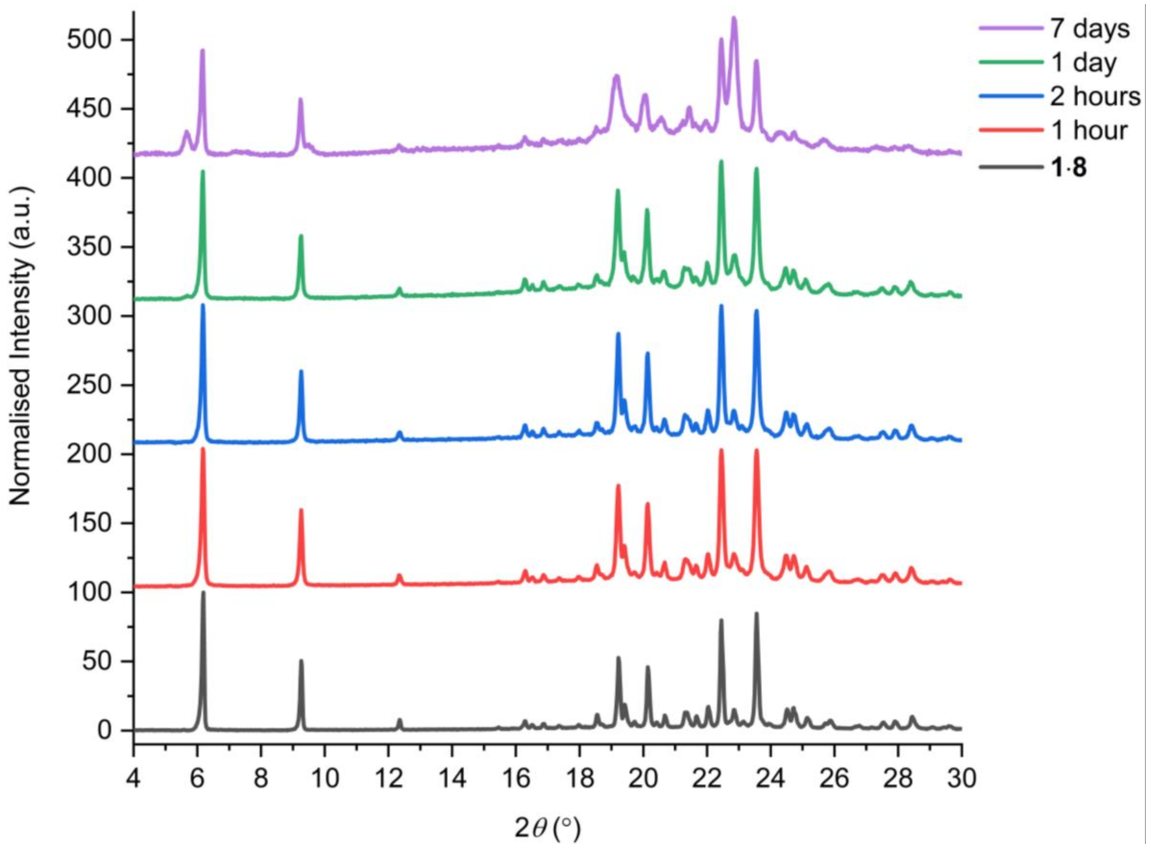

Figure S40. The experimental PXRD patterns of **1·8** irradiated for different durations by UV light at 254 nm.

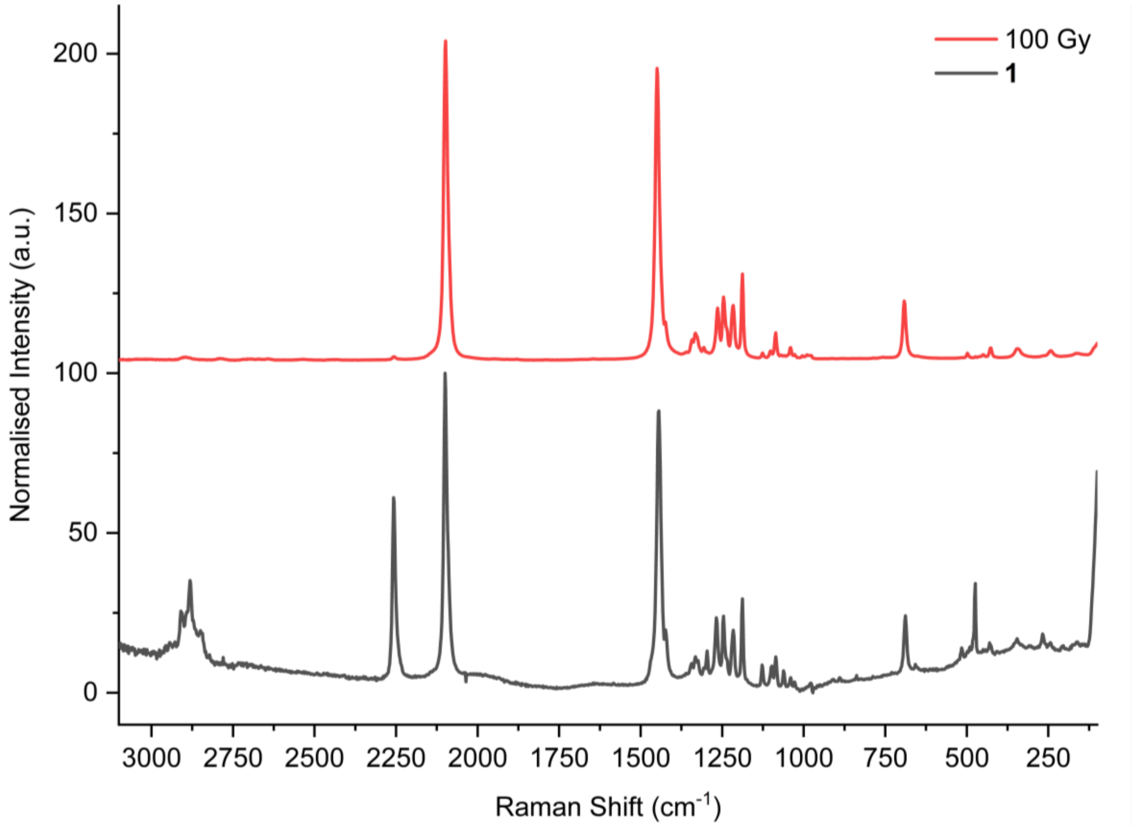

Figure S41. The Raman spectra of **1** before and after 100 Gy of X-ray radiation.

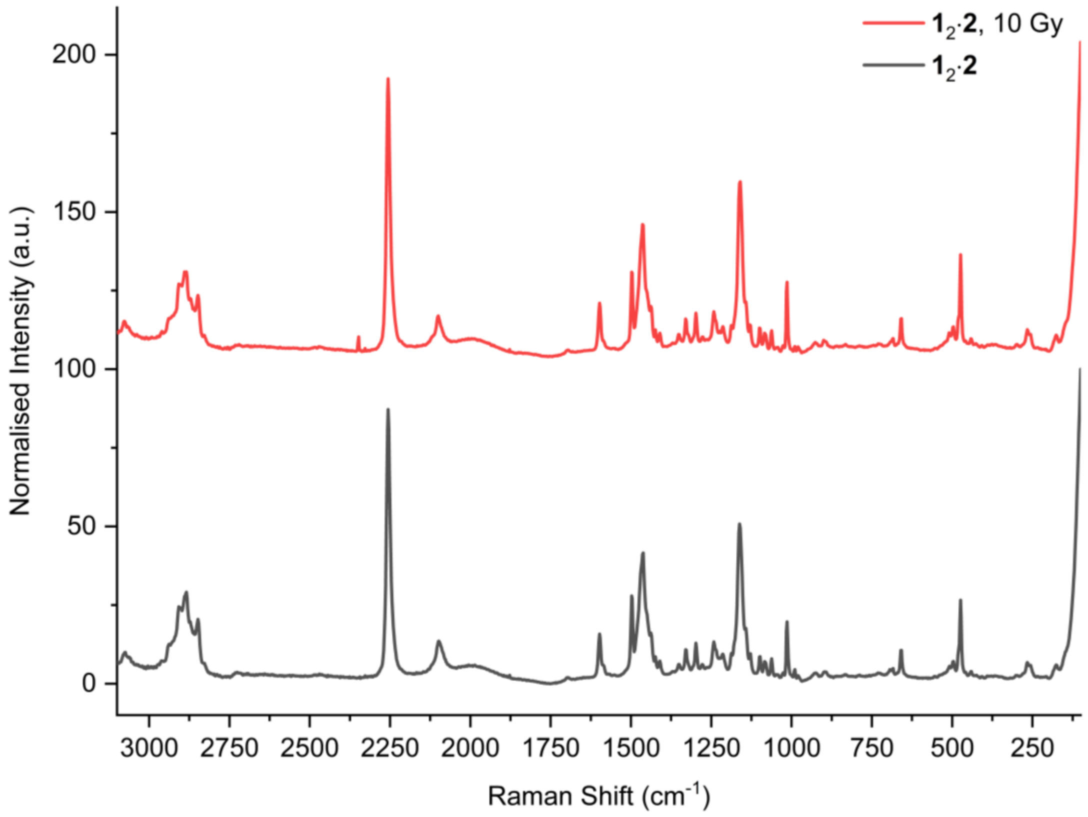

Figure S42. The Raman spectra of $\mathbf{1}_2\cdot\mathbf{2}$ before and after 10 Gy of X-ray radiation.

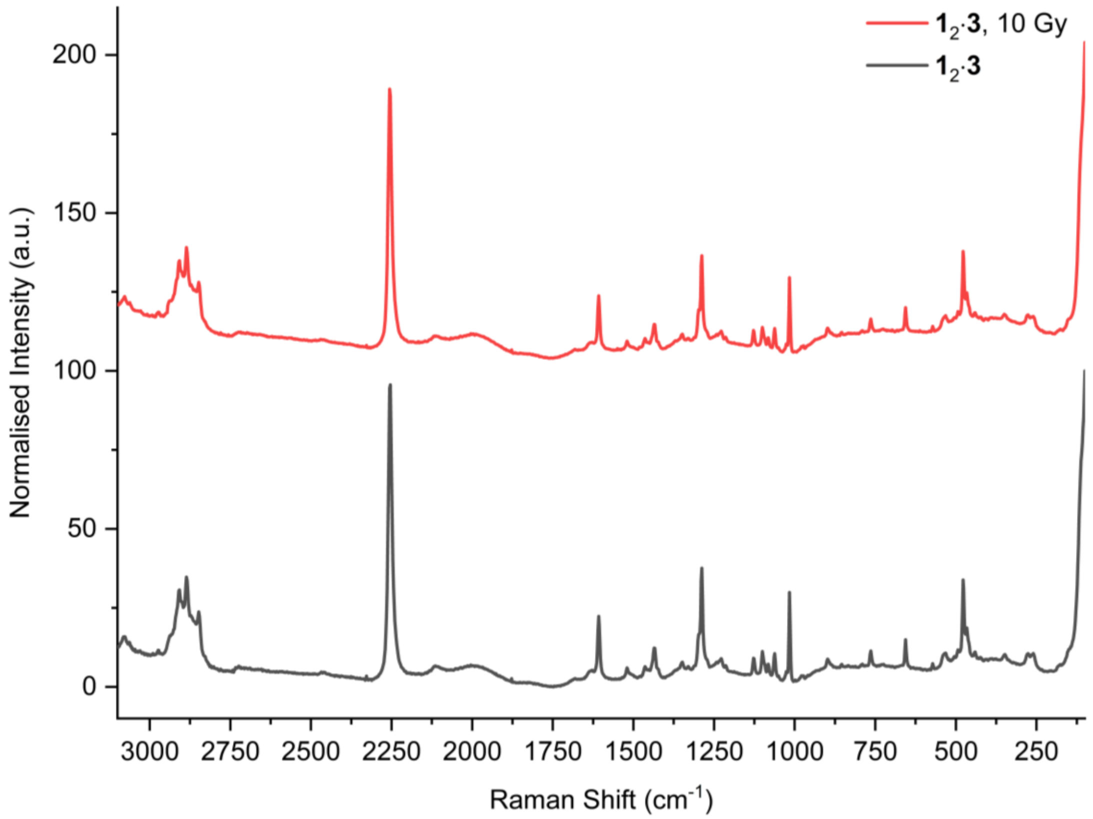

Figure S43. The Raman spectra of $\mathbf{1}_2{\cdot}\mathbf{3}$ before and after 10 Gy of X-ray radiation.

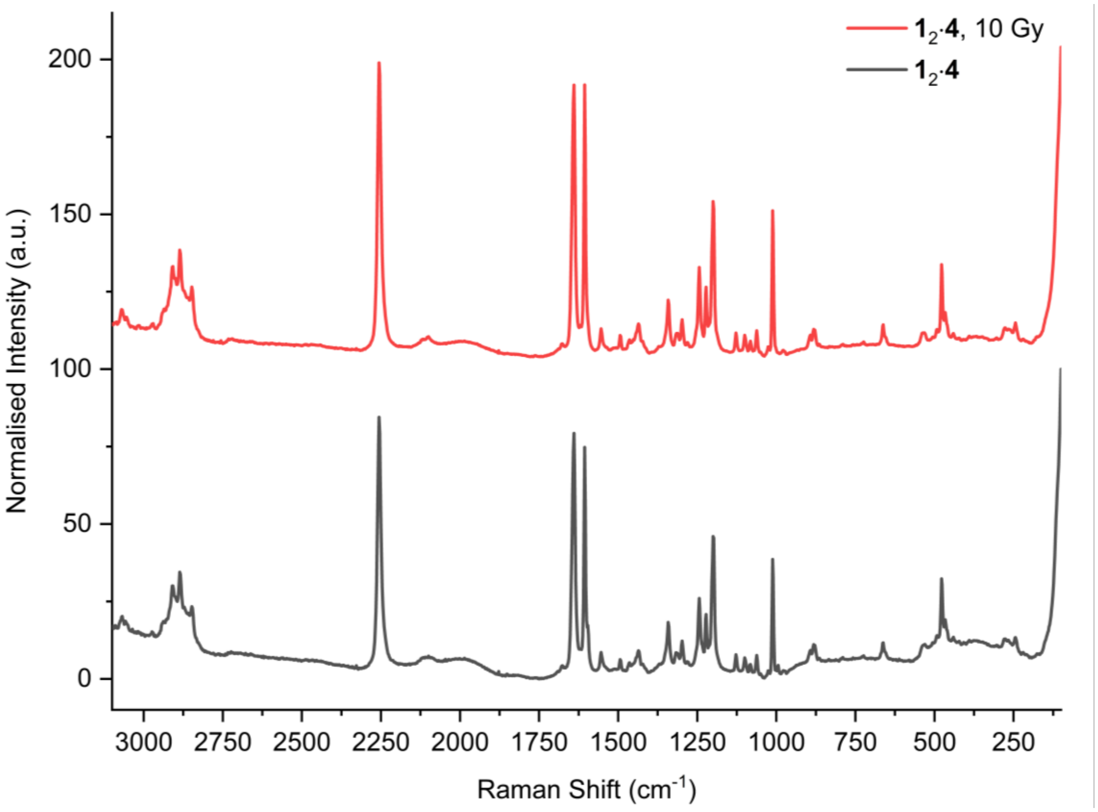

Figure S44. The Raman spectra of $\mathbf{1}_2{\cdot}\mathbf{4}$ before and after 10 Gy of X-ray radiation.

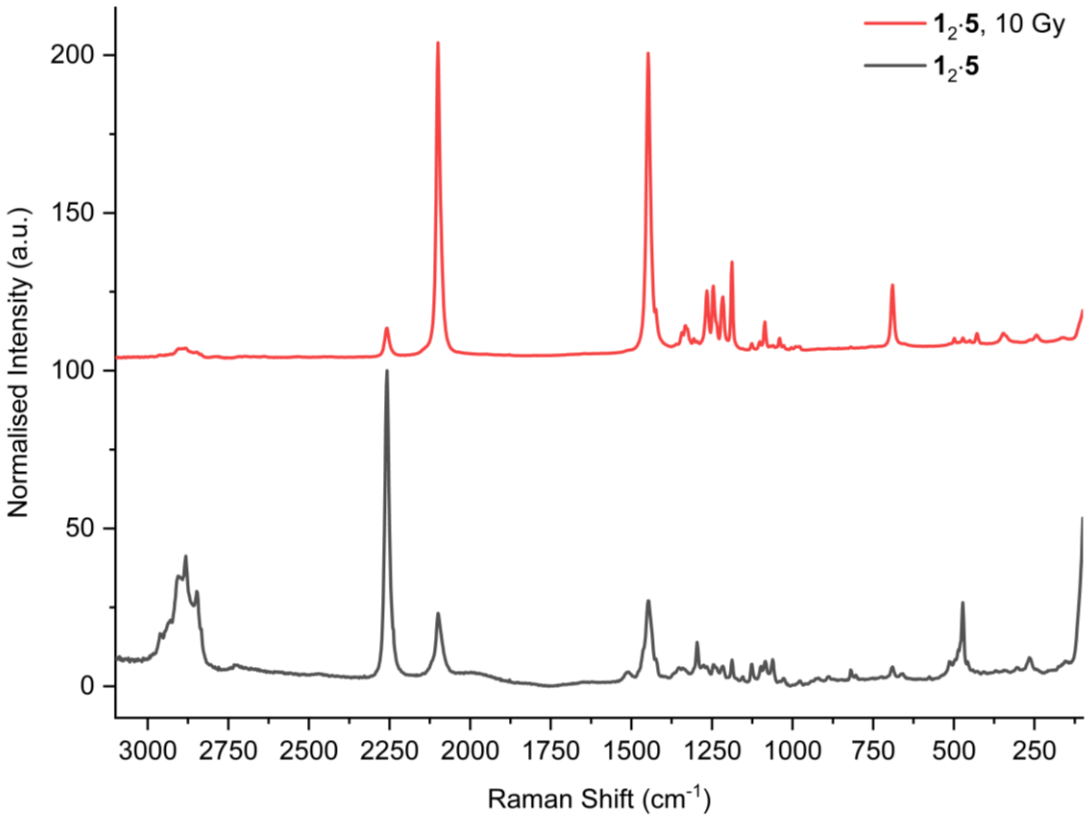

Figure S45. The Raman spectra of **1**$_2$·**5** before and after 100 Gy of X-ray radiation.

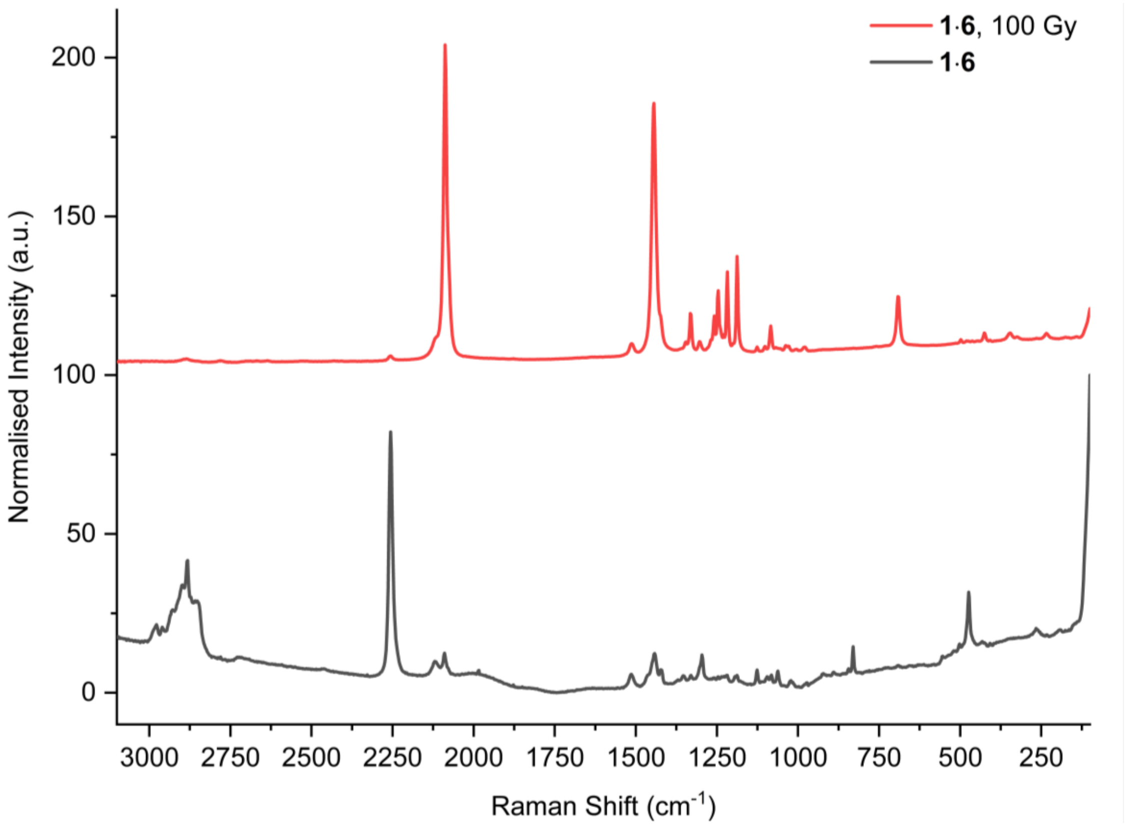

Figure S46. The Raman spectra of **1·6** before and after 100 Gy of X-ray radiation.

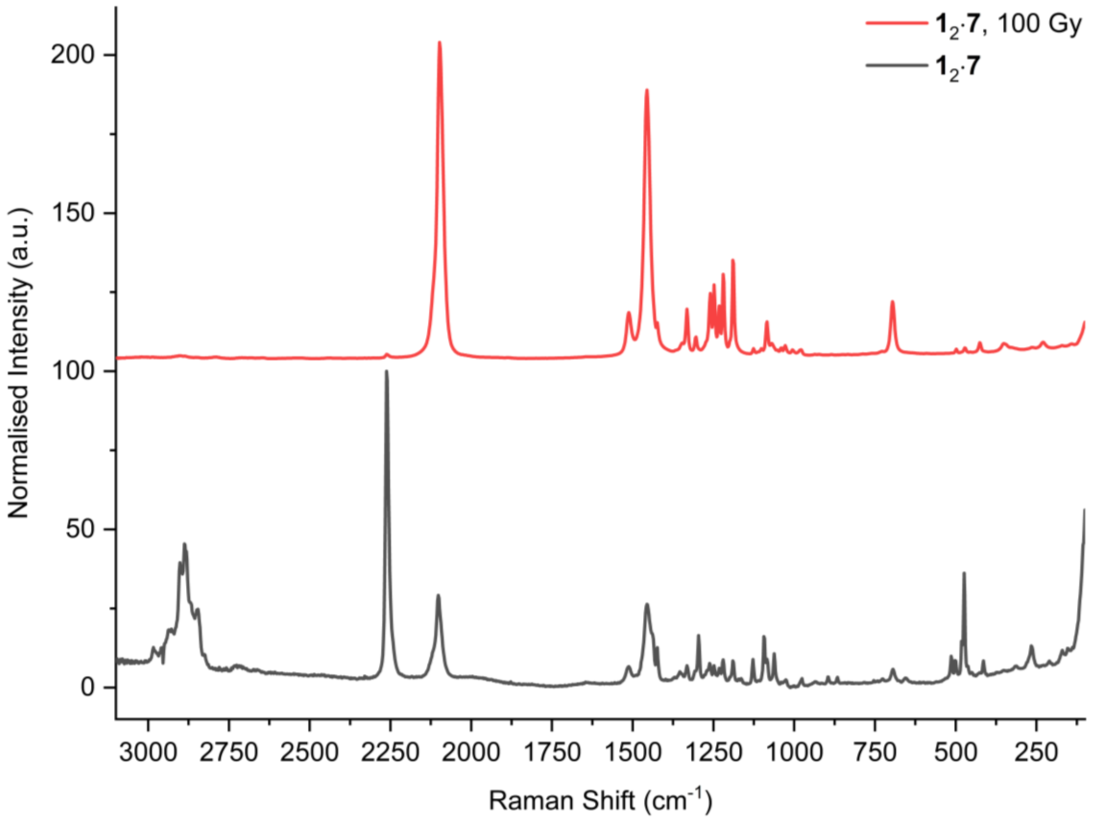

Figure S47. The Raman spectra of $\mathbf{1}_2{\cdot}\mathbf{7}$ before and after 100 Gy of X-ray radiation, highlighting the pre-resonance Raman effect by the differences in relative intensities of bands.

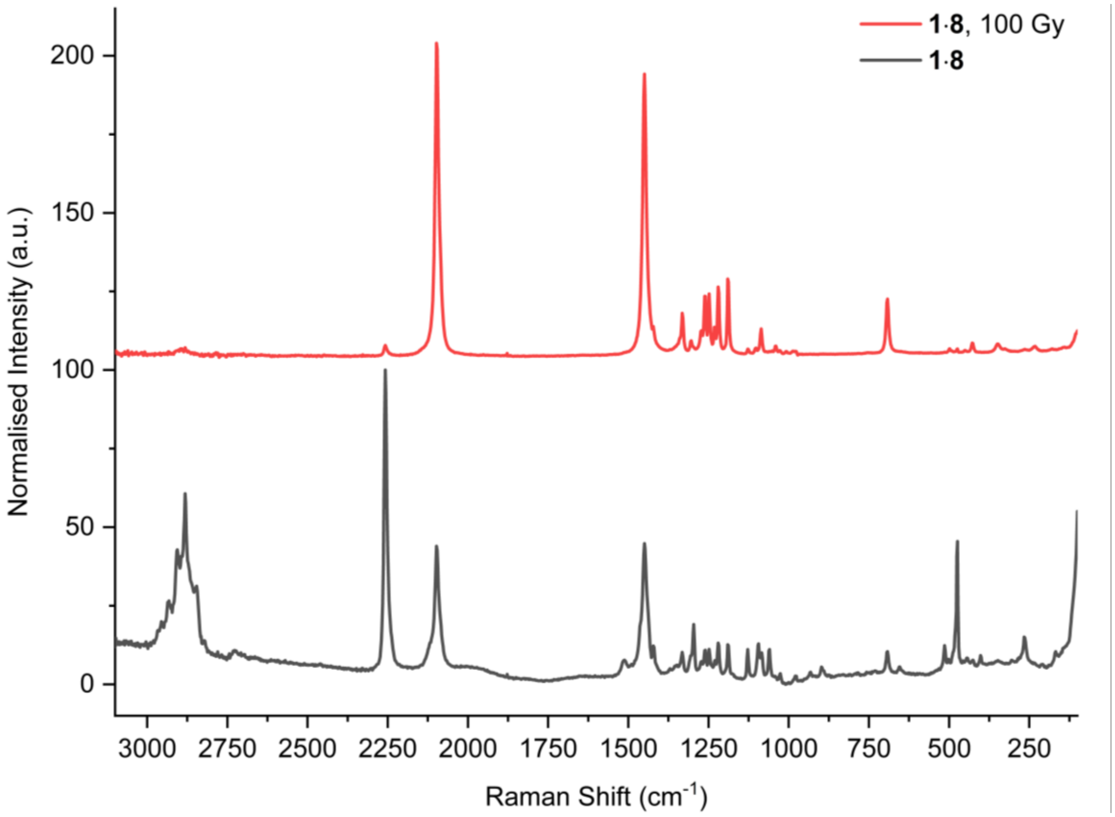

Figure S48. The Raman spectra of **1·8** before and after 100 Gy of X-ray radiation.
